# Genetic Analysis of Arrhythmogenic Diseases in the Era of NGS: The Complexity of Clinical Decision-Making in Brugada Syndrome

**DOI:** 10.1371/journal.pone.0133037

**Published:** 2015-07-31

**Authors:** Catarina Allegue, Mònica Coll, Jesus Mates, Oscar Campuzano, Anna Iglesias, Beatriz Sobrino, Maria Brion, Jorge Amigo, Angel Carracedo, Pedro Brugada, Josep Brugada, Ramon Brugada

**Affiliations:** 1 Cardiovascular Genetics Center, IdIBGi-Universitat de Girona, Girona, Spain; 2 Grupo Medicina Xenómica, Instituto de Investigación Sanitaria de Santiago de Compostela, Santiago de Compostela, Spain; 3 Fundación Pública Galega de Medicina Xenómica, SERGAS, Santiago de Compostela, Spain; 4 Center of Excellence in Genomic Medicine Research (CEGMR), King Abdulaziz University, Jeddah, Saudi Arabia; 5 Heart Rhythm Management Center, UZ Brussel-VUB, Brussels, Belgium; 6 Cardiology Unit, Hospital Clínic, Universitat de Barcelona, Barcelona, Spain; University of Tampere, FINLAND

## Abstract

**Background:**

The use of next-generation sequencing enables a rapid analysis of many genes associated with sudden cardiac death in diseases like Brugada Syndrome. Genetic variation is identified and associated with 30–35% of cases of Brugada Syndrome, with nearly 20–25% attributable to variants in *SCN5A*, meaning many cases remain undiagnosed genetically. To evaluate the role of genetic variants in arrhythmogenic diseases and the utility of next-generation sequencing, we applied this technology to resequence 28 main genes associated with arrhythmogenic disorders.

**Materials and Methods:**

A cohort of 45 clinically diagnosed Brugada Syndrome patients classified as *SCN5A*-negative was analyzed using next generation sequencing. Twenty-eight genes were resequenced: *AKAP9*, *ANK2*, *CACNA1C*, *CACNB2*, *CASQ2*, *CAV3*, *DSC2*, *DSG2*, *DSP*, *GPD1L*, *HCN4*, *JUP*, *KCNE1*, *KCNE2*, *KCNE3*, *KCNH2*, *KCNJ2*, *KCNJ5*, *KCNQ1*, *NOS1AP*, *PKP2*, *RYR2*, *SCN1B*, *SCN3B*, *SCN4B*, *SCN5A*, *SNTA1*, and *TMEM43*. A total of 85 clinically evaluated relatives were also genetically analyzed to ascertain familial segregation.

**Results and Discussion:**

Twenty-two patients carried 30 rare genetic variants in 12 genes, only 4 of which were previously associated with Brugada Syndrome. Neither insertion/deletion nor copy number variation were detected. We identified genetic variants in novel candidate genes potentially associated to Brugada Syndrome. These include: 4 genetic variations in *AKAP9* including a *de novo* genetic variation in 3 positive cases; 5 genetic variations in *ANK2* detected in 4 cases; variations in *KCNJ2* together with *CASQ2* in 1 case; genetic variations in *RYR2*, including a *de novo* genetic variation and desmosomal proteins encoding genes including *DSG2*, *DSP* and *JUP*, detected in 3 of the cases. Larger gene panels or whole exome sequencing should be considered to identify novel genes associated to Brugada Syndrome. However, application of approaches such as whole exome sequencing would difficult the interpretation for clinical purposes due to the large amount of data generated. The identification of these genetic variants opens new perspectives on the implications of genetic background in the arrhythmogenic substrate for research purposes.

**Conclusions:**

As a paradigm for other arrhythmogenic diseases and for unexplained sudden death, our data show that clinical genetic diagnosis is justified in a family perspective for confirmation of genetic causality. In the era of personalized medicine using high-throughput tools, clinical decision-making is increasingly complex.

## Introduction

In the last 20 years, the advent of advanced genetics has enabled rapid progress in the field of inherited arrhythmogenic diseases associated with sudden cardiac death (SCD). In addition, the genetic data have been rapidly translated into the clinical field, to be used in diagnostic, risk stratification, and therapeutic strategies. Although genetics has been advocated as a tool to be interpreted in the context of the clinical phenotype as a complementary test, in the field of arrhythmogenic diseases genetics is emerging as a diagnostic tool in itself, providing a clue to unexplained death. Herein, genetics is not a complement, but a substitute for the unsuccessful clinical and forensic investigation [1]. Thus, in recent years we have moved from the use of genetics to confirm a known disease, to the use of genetics to diagnose an unknown disease.

The recent developments in massively parallel sequencing (next-generation sequencing, NGS) have provided access to thorough genetic screening. This technology is rapidly transitioning to clinical practice, adding a new level of complexity to patient diagnosis and care. NGS allows a fast and cost-effective approach for the genetic screening of a large panel of genes, representing an ambitious strategy compared with Sanger sequencing. When combined with inquiry into large-scale international genetic databases, NGS allows the scientific community to acquire deeper spectra of the genetic variants contributing to specific phenotypes. However, its use in clinical diagnosis remains a challenge due to the large amount of genetic information that has to be interpreted to evaluate its pathological relevance.

NGS is beginning to be applied to arrhythmogenic diseases like Brugada syndrome (BrS). The BrS is a rare, inherited arrhythmogenic disorder characterized by the presence of ST-segment elevation in the right precordial leads (V_1_ to V_3_), referred to as electrocardiogram (ECG) type I. BrS causes SCD in the structurally normal heart, and affects predominantly men during the third and fourth decade of life. Although the disease has classically been described as a primary electrical disorder involving the sodium channel and leading to the characteristic ECG, it has been proposed that BrS may actually encompass a heterogeneous group of disorders with a variety of genetic and clinical phenotypes. Currently, the major genetic contribution to BrS is variation in the *SCN5A* gene, which represents 20_25% of all clinically diagnosed BrS cases. Another 15 genes have been associated with the disease, but together represent only about 5_10% of BrS cases [2]. Thus, close to 70% of BrS cases remain unexplained after comprehensive genetic analysis of all previously associated genes. Current guidelines recommend to genetically screening restricted to the *SCN5A* gene as the most resolute and cost-effective strategy for genetic diagnosis of Brugada Syndrome affected patients [3][4]

To evaluate the role of genetic variants in arrhythmogenic diseases, we used NGS technology to resequence 28 main genes associated with arrhythmogenic SCD in a cohort of BrS patients. The results allow us to assess the value of this approach in confirming a diagnosis of a clear clinical phenotype and also to draw conclusions on its potential value in forensic investigation of patients with unexplained SCD.

## Materials and Methods

### Patient recruitment

We retrospectively recruited a total of 45 samples/clinical histories of adult patients clinically diagnosed with BrS in a multicenter effort. These samples serve as the index case in each family. All index cases included in the study were negative for pathogenic genetic variants in *SCN5A* (see below). Informed consent for all samples was obtained in accordance with local institutional review board guidelines of the Hospital Josep Trueta (Girona, Spain) and conforms to the principles outlined in the Declaration of Helsinki. The project was approved by the local review board (Hospital Josep Trueta-Girona). All patients and relatives signed their written consent for research purposes before participation in this study. The diagnosis of BrS was accepted when the patients showed an ECG type 1 (basally or after the administration of intravenous sodium channel blockers) in conjunction with at least one clinical criterion, reflecting the occurrence of documented ventricular arrhythmia, family history (of SCD or BrS), and/or symptoms secondary to arrhythmia. Structural heart disease was ruled out in all participating individuals. S2 Fig and S3 Fig show two examples of ECG type 1 observed in two of the cases included at the study.

### Target enrichment: Custom resequencing panel

At the moment of the study, we had genetically analyzed a total of 63 individuals for *SCN5A* by conventional Sanger sequencing: 18 of them resulted genetically positive for *SCN5A* (29% of the cases) and the remaining 45 cases (71% of the cases) remained genetically negative. These percentages are in agreement with data described in the literature and guidelines. In all these 45 samples, neither pathogenic nor potentially pathogenic variations were detected after Sanger sequencing of *SCN5A*. All 45 BrS cases were genetically evaluated by means of target resequencing of exonic, UTR, and intron-exon boundary regions of 28 core genes related to SCD, included in a panel of massively parallel sequencing in SOLID 4 platform (Life Technologies Waltham, MA USA). This panel includes *AKAP9*, *ANK2*, *CACNA1C*, *CACNB2*, *CASQ2*, *CAV3*, *DSC2*, *DSG2*, *DSP*, *GPD1L*, *HCN4*, *JUP*, *KCNE1*, *KCNE2*, *KCNE3*, *KCNH2*, *KCNJ2*, *KCNJ5*, *KCNQ1*, *NOS1AP*, *PKP2*, *RYR2*, *SCN1B*, *SCN3B*, *SCN4B*, *SCN5A*, *SNTA1*, and *TMEM43*. We included genes associated with cardiac channelopathies as well as genes associated with arrhythmogenic right ventricular cardiomyopathy (ARVC), a disease difficult to diagnose in its incipient forms even with powerful imaging techniques (Table 1).

**Table 1:** Specific Ensemble isoforms and RefSeq codes included in the target resequencing custom panel. The design interrogates 191.12 Kb of enriched genomic regions.

Gene ID	Ensembl Transcript ID	RefSeq	n Exons	Associated with BrS (%)
*AKAP9*	ENST00000356239	NM_005751	50	No
*ANK2*	ENST00000357077	NM_001148	46	No
*CACNA1C*	ENST00000347598	NM_001129827	47	Yes (<5%)
*CACNB2*	ENST00000396576	NM_000724	13	Yes (<5%)
*CASQ2*	ENST00000261448	NM_001232	11	No
*CAV3*	ENST00000343849	NM_033337	2	No
*DSC2*	ENST00000280904	NM_024422	16	No
*DSG2*	ENST00000261590	NM_001943	15	No
*DSP*	ENST00000379802	NM_004415	24	No
*GPD1L*	ENST00000282541	NM_015141	8	Yes (<5%)
*HCN4*	ENST00000261917	NM_005477	8	Yes (<5%)
*JUP*	ENST00000393931	NM_002230	14	No
*KCNE1*	ENST00000337385	NM_001270402;NM_001270403	3	No
*KCNE2*	ENST00000290310	NM_172201	2	No
*KCNE3*	ENST00000310128	NM_005472	3	Yes (<5%)
*KCNH2*	ENST00000262186	NM_000238	15	No
*KCNJ2*	ENST00000243457	NM_000891	2	No
*KCNJ5*	ENST00000338350	NM_000890	4	No
*KCNQ1*	ENST00000155840	NM_000218	16	No
*NOS1AP*	ENST00000361897	NM_001164757	10	No
*PKP2*	ENST00000070846	NM_004572	14	Yes (<5%)
*RYR2*	ENST00000366574	NM_001035	105	No
*SCN1B*	ENST00000415950	NM_199037	3	Yes (<5%)
*SCN3B*	ENST00000392770	NM_018400	6	Yes (<5%)
*SCN4B*	ENST00000324727	NM_174934	5	No
*SCN5A*	ENST00000333535	NM_198056	28	Yes (20–25%)
*SNTA1*	ENST00000217381	NM_003098	8	No
*TMEM43*	ENST00000306077	NM_024334	12	No

The custom design of the Resequencing Panel is explained in S1 Methods. The 28 genes were enriched using SureSelect Custom Target Enrichment System kit (Agilent Technologies, Santa Clara_CA USA) following manufacturing protocol "SureSelect Target Enrichment System for SOLiD Fragment and Paired-End Sequencing version 1.3.” (See S1 Methods for custom design of the resequencing panel and the bioinformatics pipeline explanation and S1 Fig for sequencing statistics)

The median percentage of reads on target was 46% for all 45 samples (ranging from 43% to 52%). The median coverage was 129x (ranging from 100x to 179x), and the 25th, 75th, and 95th percentiles were 83x, 216x, and 469x, respectively. (See S1, S2 and S3 Tables)

### Variant validation and familial segregation study

Potential pathogenic rare variants with a MAF (minor allele frequency) under 1% were consulted in locus-specific databases that included dbSNP [5], Ensembl genome browser [6], the 1000 Genomes Project [7], and EVS (Exome Variant Server, NHLBI GO Exome Sequencing Project _ESP).

All genetic variants detected in the index cases were validated by conventional Sanger sequencing. Previously published function-affecting variants as well as potential pathogenic genetic variants were also analyzed in a total of 85 relatives by direct sequencing of those genomic positions.

*In silico* analysis of pathogenicity of rare nonsynonymous variants was performed with Condel Analysis [8] Mutation Taster (http://www.mutationtaster.org/), Polyphen (http://genetics.bwh.harvard.edu/pph2/), and Provean (http://provean.jcvi.org/) (Table 2 and S1 Methods). Data related to genetic variants detected in the study were submitted to the Leiden Open Variation Database 3.0 (URL http://www.lovd.nl/3.0/home) (Table 2). We have recently published a restrictive score to consider the potential pathogenicity of a genetic variant for clinical assessment [9]. This score was applied to the variants presented in this article (Table 2)

**Table 2:** Report of rare variants detected. NA: DNA not available from relatives. IP: Incomplete penetrance. CM: Human gene variation database code. MAF: Minor allele frequency in the NHLBI Exome Sequencing Project (ESP). LOVD ID: Submission ID on Leiden Open Variation Database. EA: European American population. AA: African American population. Last revised January 2015. Predictors: C: Condel; MT: Mutation Taster; PPH2: Polyphen; Prov: Provean. N:N eutral; D: Deleterious; P: Polymorphism; DC: Disease causing; B: Benign; PD: Possibly_Damaging pathogenicity score based in Campuzano et al. score [9] and applied to BrS. VUS: Variant of uncertain significance.

Group	Index case #LOVD ID	Gene	Variant	Variant code/De novo Detected	MAF % EA	MAF % AA	MAF % All	CONDEL score	Prediction C/MT/PPH2/Prov	Segregation	Pathogenicity score
Group 1	1 #00028976	ANK2	*c*.*8843C>G (p*.*(Ala2948Gly))*	rs138438183	0.0349	0.0227	0.0308	0.003	N/P/B/N	Yes	VUS
	1 #00028976	PKP2	*c*.*1577C>T (p*.*(Thr526Met))*	rs146882581 (CM113820)	0.2326	0.6355	0.3691	0.084	N /P/B/N	No	Benign
	2#00028977	ANK2	*c*.*7132G>A (p*.*(Glu2378Lys))*	rs141191319	0.3488	0.0227	0.2384	0.003	N/P/B/N	No	Benign
	2#00028977	ANK2	*c*.*7334A>G (p*.*(Asp2445Gly))*	-				0.724	D/DC/PD/D	Yes	VUS
	2#00028977	AKAP9	*c*.*5246T>C (p*.*(Ile1749Thr))*	rs150016098	0.0465	0.0227	0.0384	0.950	D/DC/PD/D	No	Benign
	3 #00028978	CASQ2	*c*.*1148A>G (p*. *(Asp383Gly))*	rs397516640	-	-	-	0.01	N/P/B/D	Yes	VUS
	3 #00028978	CACNB2	*c*.*1511C>T (p*.*(Thr504Ile))*	rs143326262	0.2093	0.0227	0.1461	0.011	N/DC/PD/N	No	Benign
	3 #00028978	PKP2	*c*.*1577C>T (p*.*(Thr526Met))*	rs146882581(CM113820)	0.2326	0.6355	0.3691	0.084	N/P/B/N	No	Benign
	3 #00028978	KCNJ2	*c*.*532G>A (p*.*(Ala178Thr))*	-				0.005	N/DC/PD/N	Yes	VUS
	4 #00028979	HCN4	*c*.*3577G>C (p*. *(Glu1193Gln))*	rs200507617	0.0233	0	0.0154	0.709	D/DC/B/N	Yes	VUS
	5 #00028980	JUP	*c*.*475G>T (p*.*(Val159Leu))*	-				0.019	N/DC/PD/D	Yes	VUS
	6 #00028981	RYR2	*c*.*3803T>C (p*.*(Ile1268Thr))*	De novo				0.508	D/DC/B/D	Yes	VUS
	7 #00028982	AKAP9	*c*.*3827G>A (p*.*(Arg1276Gln))*	rs146797353	0.8856	0.318	0.6932	0.007	N/P/B/N	IP	VUS
	7 #00028982	AKAP9	*c*.*8573A>G (p*.*(Tyr2858Cys))*	De novo				0.007	N/P/B/D	IP	VUS
	8 #00028983	AKAP9	*c*.*8656A>G (p*.*(Ile2886Val))*	rs143283097	0.035	0	0.0231	0.003	N/P/B/N	IP	VUS
	9 #00028984	ANK2	*c*.*3914G>A (p*. *(Arg1305Gln))*	-				0.981	D/DC/PD/N	IP	VUS
	9 #00028984	PKP2	*c*.*1781T>C (p*.*(Ile594Thr))*	-				0.974	D /DC/PD/D	No	Benign
	10 #00028985	DSP	*c*.*1150G>C (p*. *Glu384Gln)*	-				0.766	D/DC/PD/N	IP	VUS
	11 #00028986	DSP	*c*.*5218G>A (p*.*(Glu1740Lys))*	rs142885240	0.1279	0.0227	0.0923	0.408	N/P/PD/N	IP	VUS
	12 #00028987	PKP2	*c*.*2504A>G (p*.*(Lys835Arg))*	rs372729739	0.0116	0	0.0077	0.254	N/DC/PD/N	IP	VUS
Group 2	13 #00028988	ANK2	*c*.*5758G>A (p*.*(Gly1920Arg))*	rs140189724	0.0233	0.0227	0.0231	0.001	N/P/PD/N	NA	VUS
	14 #00028996	CACNA1C	*c*.*5875G>C (p*.*Gly1959Arg)*	-				0.003	N/DC/PD/N	NA	VUS
	15 #00028990	DSG2	*c*.*3209C>T (p*.*(Thr1070Met))*	rs149617776	0.0604	0.0265	0.0498	0.019	N/P/B/N	NA	VUS
	16 #00028992	DSP	*c*.*8455A>C (p*. *(Met2819Leu))*	rs138329459 (CM132698)	0.0116	0.0681	0.0308	0.015	N/P/B/N	NA	VUS
	17 #00028993 #00028994	RYR2	*c*.*5056C>T (p*. *(Leu1686Phe))*	-				0.949	D/DC/PD/N	NA	VUS
	17 #00028993 #00028994	PKP2	*c*.*302G>A (p*. *(Arg101His))*	rs149542398	0.0465	0.0227	0.0384	0.732	D/P/B/N	NA	VUS
Group 3	18 #00028995	ANK2	*c*.*2945G>A (p*. *(Arg982Gln))*	-				0.981	D/DC/PD/D	No	Benign
	19 #00028997	CACNA1C	*c*.*2449C>T (p*.*(Pro817Ser))*	rs112532048	0.4679	0.0738	0.3387	0.004	N/DC/PD/N	No	Benign
	20 #00028998	CACNB2	*c*.*1925T>C (p*.*(Ile642Thr))*	-				0.02	N/DC/B/N	No	Benign
	21 #00028999	DSG2	*c*.*1003A>G (p*.*(Thr335Ala))*	rs191564916	0.061	0	0.0419	0.287	N/P/PD/N	No	Benign
	22 #00029000	DSG2	*c*. *473T>G (p*. *(Val158Gly))*	rs191143292 (CM070921)	0.7881	0.2403	0.617	0.867	D /DC/B/D	No	Benign

## Results and Discussion

### Clinical profiles

Our cohort included 45 families (130 individuals, 45 index cases plus 85 relatives). The average age at the time of diagnosis of our index cases was 43.5 ± 14.83 years old; 31/45 (68.9%) were males. Basal type 1 ECG was present in 27/45 (60%), and the remaining cases showed positive ECG after drug test (flecainide, ajmaline, or procainamide). Of all index cases, 22/45 (55.6%) had a previous clinical history of BrS in the family, and 19/45 (42.2%) had suffered previous syncope, seizures, or nocturnal agonal respiration. Familial history of SCD occurred in 19/45 (42.2%) of our families. All the individuals under genetic analysis were clinically evaluated using the same diagnostic procedures following international guidelines.

### Genetic results

Thirty genetic variants, all of them in exonic regions of 12 genes, were detected in the heterozygous state in 22 index cases (Table 3). In the remaining 23 cases, no potentially pathogenic variation was identified. The average age of our 22 index cases was 46.09±13.89 years old, with 14

**Table 3:** Relationship of genes in which rare variation was detected. Segregation study outcomes are shown. BrS: Brugada syndrome, LQTS: Long QT syndrome, ATS: Anderson-Tawil syndrome. CPVT: catecholaminergic polymorphic right ventricular tachycardia. SSS: Sick Sinus syndrome. *Cerrone *et al*. recently defined the co-existence of clinical BrS and genetic variations in *PKP2* [10].

Gene	Ensembl Isoform	RefSeq	Positive segregation study	Previously associated with BrS	Associated disease
*AKAP9*	ENST00000356239	NM_005751	Yes	No	LQTS11
*ANK2*	ENST00000357077	NM_001148	Yes	No	LQTS4
*CACNA1C*	ENST00000347598	NM_001129827	Unknown	Yes	LQT8, ATS1
*CACNB2*	ENST00000324631	NM_000724	No	Yes	BrS
*CASQ2*	ENST00000261448	NM_001232	Yes	No	CPVT
*DSG2*	ENST00000261590	NM_001943	No	No	Cardiomyopathy
*DSP*	ENST00000379802	NM_004415	Yes	No	Cardiomyopathy
*HCN4*	ENST00000261917	NM_005477	Yes	Yes	SSS
*JUP*	ENST00000562805	NM_002230	Yes	No	Cardiomyopathy
*KCNJ2*	ENST00000243457	NM_000891	Yes	No	CPVT
*PKP2*	ENST00000070846	NM_004572	Yes	Yes*	Cardiomyopathy
*RYR2*	ENST00000366574	NM_001035	Yes	No	CPVT, LQTS

(63.6%) being male. Fourteen index cases showed a BrS basal type 1 ECG. Clinical data are shown in S4 Table _Clinical and familial information.

### Segregation studies

**Fig 1:**
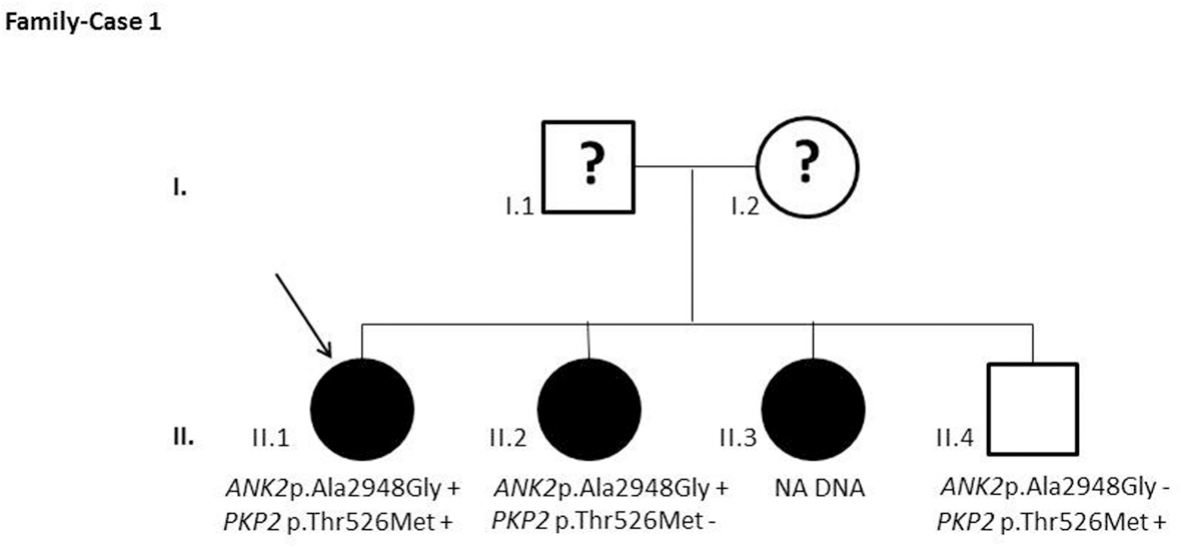
Case-Family 1. Segregation of the *ANK2* c.8843C>G (p.(Ala2948Gly)) (rs138438183) and lack of segregation on *PKP2* c.1577C>T (p.(Thr526Met)) (rs146882581_CM113820[11]), rare variant also detected in case 3. Case 1 (II.1), 47-year-old woman. Individuals (II.2 and II.3) were also diagnosed with BrS both with previous syncope and one of them (II.2) showed a positive ajmaline test.

After segregation and bioinformatic analyses, several potentially pathogenic rare variants were identified in the 22 cases. These variants would be labeled as potentially causative in the event of a screening performed in the single individual, victim of a cardiac arrest, or as part of the forensic investigation of unexplained sudden death. Multiple genetic variants were detected in 6 cases (Table 2). Clinical data and family segregation are summarized in S4 Table. Family pedigrees are shown in Fig 1 to Fig 12. These figures show the phenotype of relatives as positive or negative for BrS diagnosis. Clinical information of relatives is essential information to ascertain the pathogenicity of the genetic variant that was detected in each index case after NGS. Taking these data into account, we proceeded to divide the groups into three main classes according to segregation variable:

Figs 1–12 show Pedigrees for phenotype correlation analysis on samples in Group 1.

**Fig 2:**
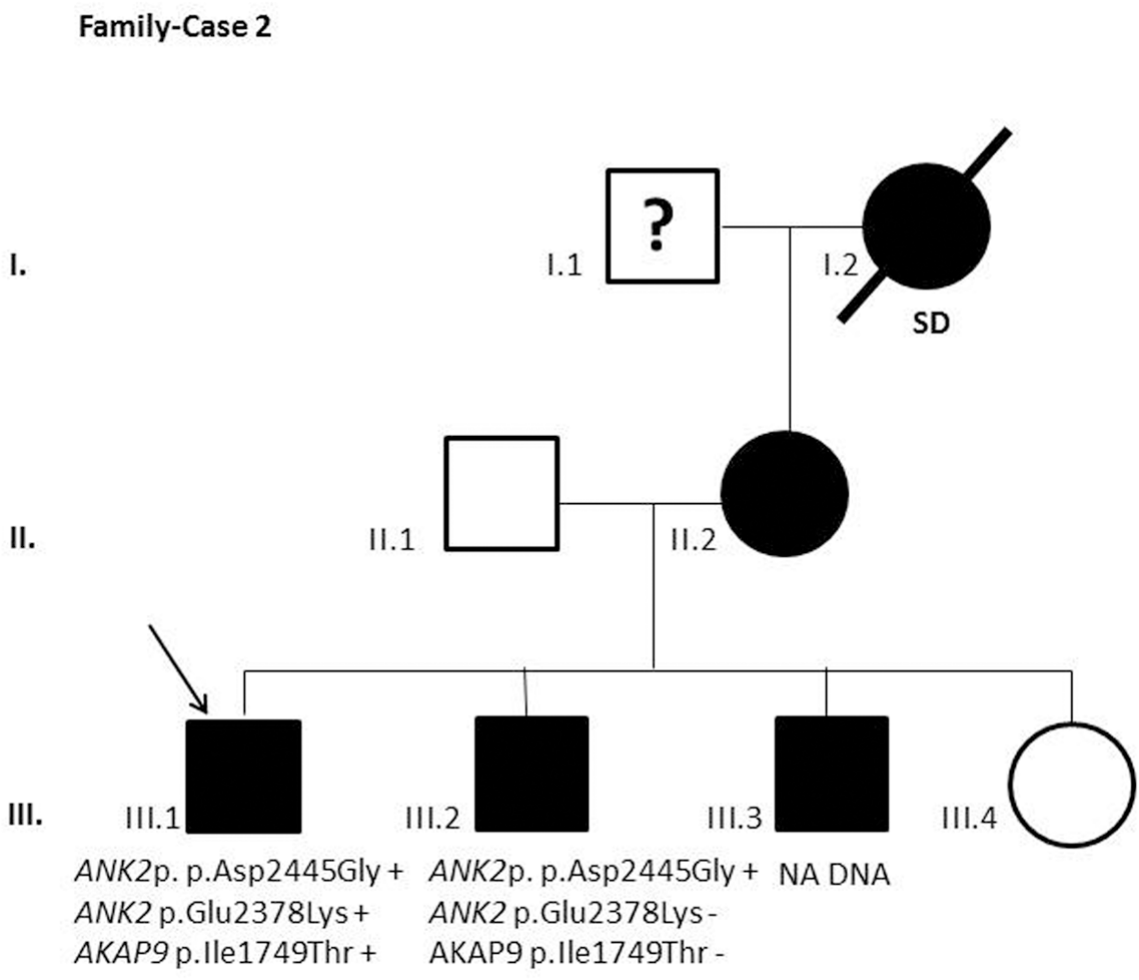
Case-Family 2. Positive segregation of *ANK2* c.7334A>G (p.Asp2445Gly) and lack of segregation on *ANK2* c.7132G>A (p.(Glu2378Lys)) (rs141191319) and *AKAP9* c.5246T>C (p.(Ile1749Thr)) (rs150016098). The family history includes two brothers diagnosed at an age of 36 and 34 (III.2 and III.3, respectively), his asymptomatic sister (III.4), and his mother (II.2) affected by BrS. Individual III.2 showed a positive ECG pattern and a positive EPS although remaining asymptomatic. An ICD was implanted. Individual III.3 had a positive ajmaline test. Their previously asymptomatic mother (II.2) showed a positive EPS and an ICD was implanted.

**Fig 3:**
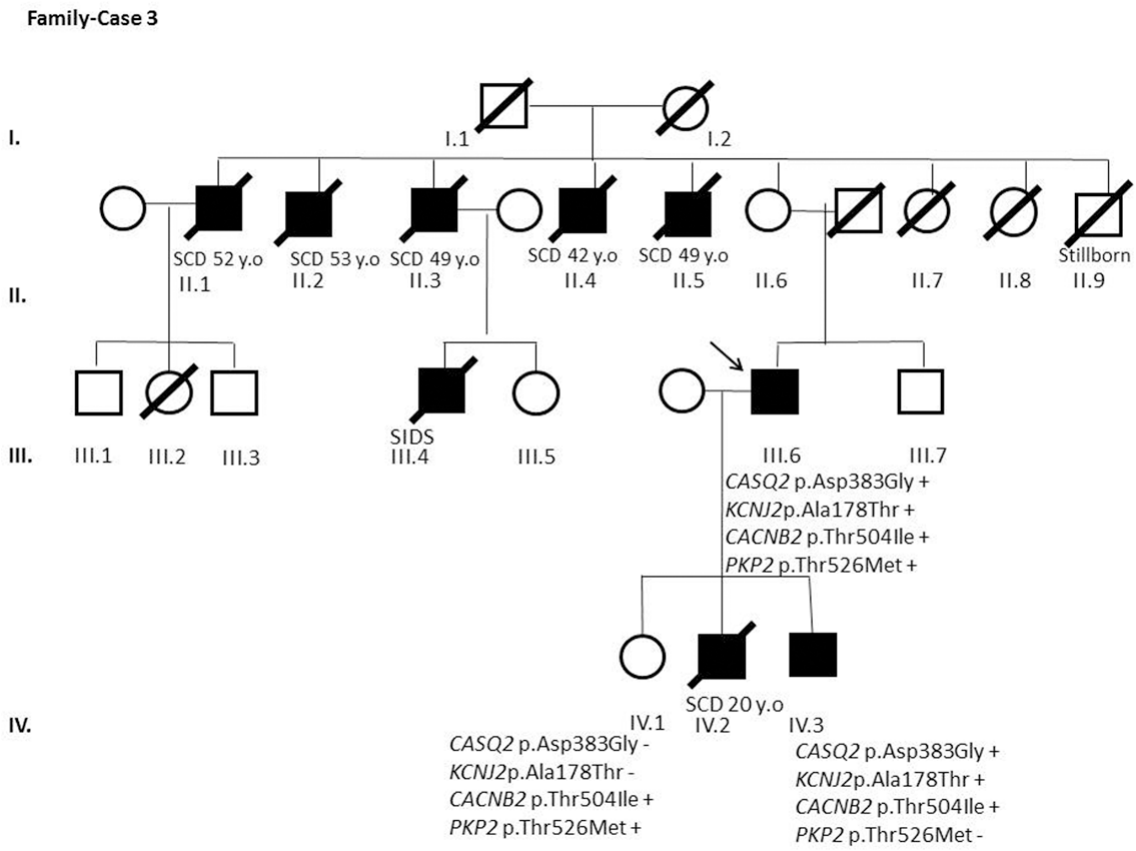
Case-Family 3. *CASQ2* c.1148A>G (p.(Asp383Gly)) and *KCNJ2* c.532G>A (p.(Ala178Thr)) segregation is shown. Also the lack of segregation of *CACNB2* c.1511C>T (p.(Thr504Ile)) (rs143326262) and *PKP2* c.1577C>T (p.(Thr526Met)) (rs146882581 also detected in Case 1). Individual I.1 died at age 65 because of chronic cardiovascular disease. Individual I.2 died at age 76 because of congestive heart failure. The second generation of this family includes 9 siblings (II.1-II.9), 5 of them-all male- died suddenly at ages between 42 and 53 years (II.1-II.5) and also a brother who died stillborn (II.9). Two siblings (II.7 and II.8), both female, died from other causes. The last sibling, a 91-year-old woman (II.6), is still alive and was diagnosed with atrial fibrillation (AF) at a young age. The third generation includes our index case (III.6), a 66-year-old man who was diagnosed with AF at 18 years of age. Notably, one SIDS case (III.4) was recorded in this generation. Clinical history and evaluation of the index case includes syncope at age 54, BrS pattern at ECG, and positive EPS, leading to an ICD implantation. His offspring are a 39-year-old asymptomatic daughter (IV.1) with negative EPS and procainamide test, a 20-year-old man (IV.2) who died suddenly, and a 31-year-old man (IV.3) diagnosed with AF, with negative procainamide test, positive EPS, and previously asymptomatic.

**Fig 4:**
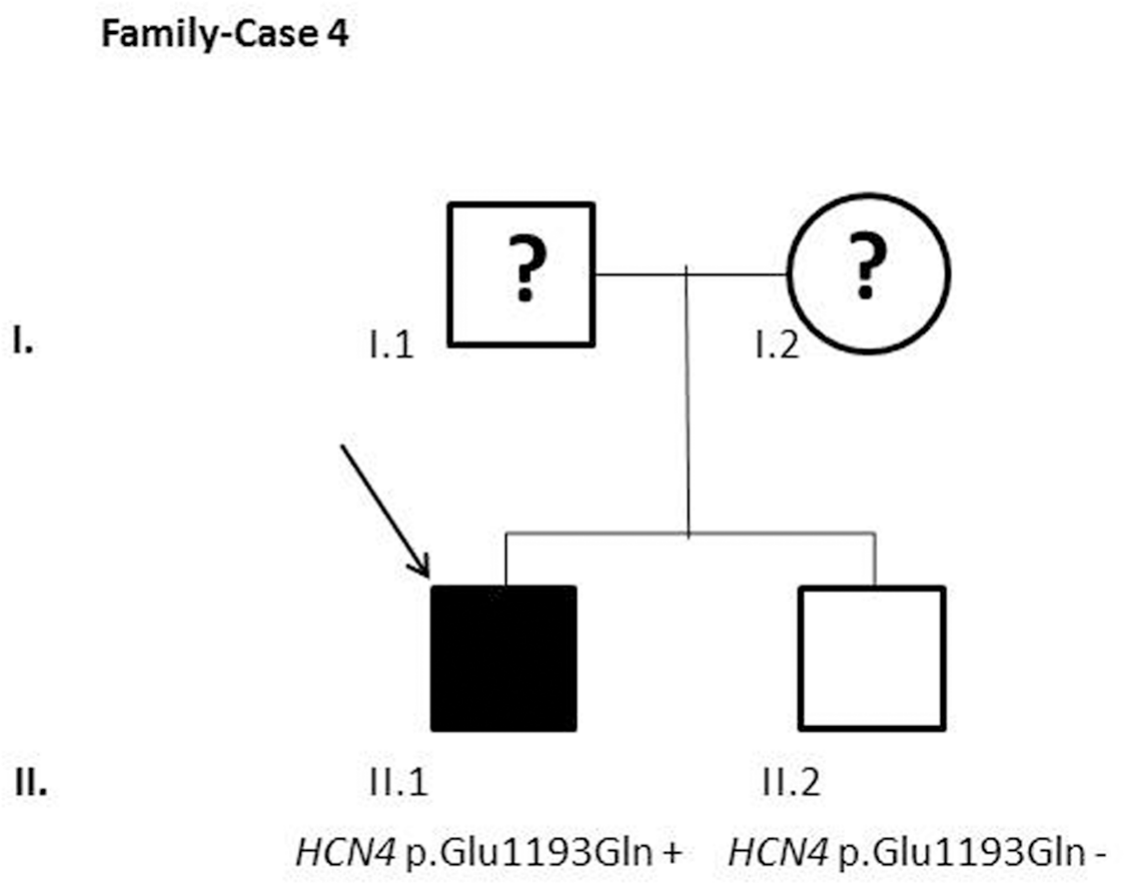
Case-Family 4. Positive segregation of *HCN4* c.3577G>C (p.(Glu1193Gln)) (rs200507617) with the pathology in the family. Individual II.1 is a symptomatic 70-year-old man (II.1) who showed a pathologic BrS ECG pattern and positive EPS, and had an ICD implanted. The genetic variant, predicted as deleterious, was absent in his healthy brother (II.2), with a non-pathological ECG.

**Fig 5:**
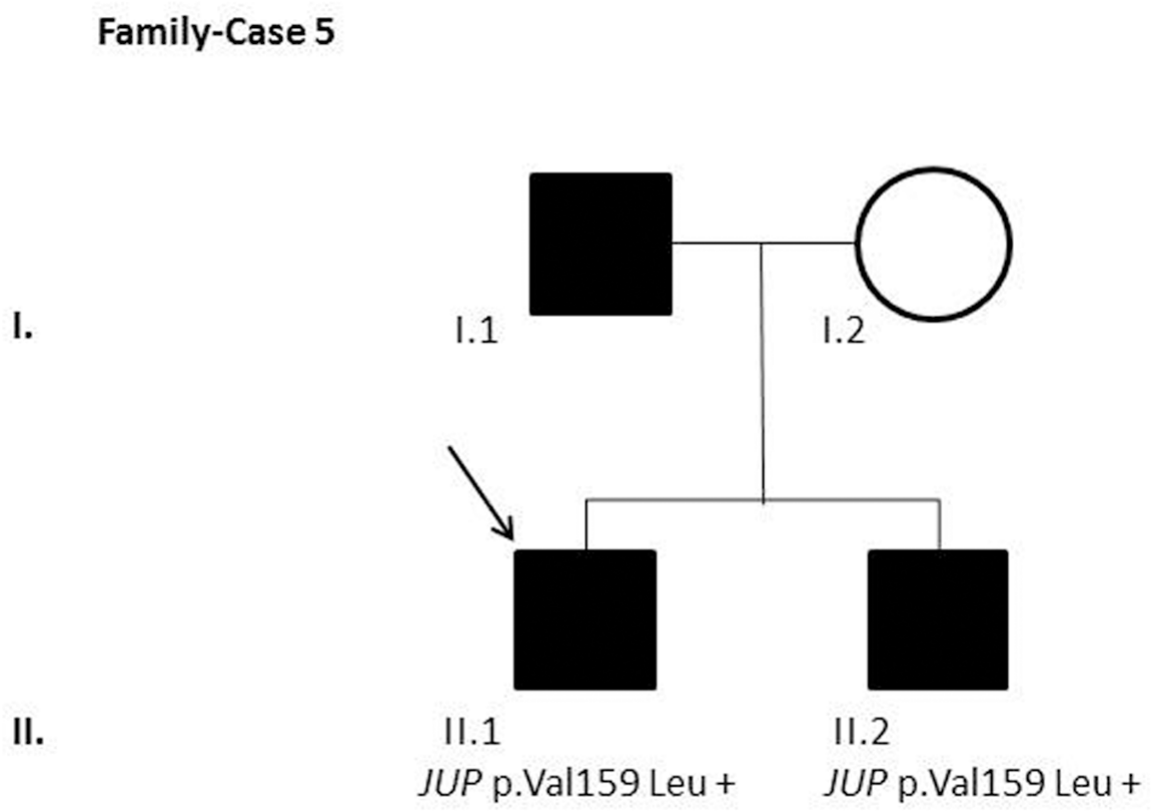
Case-Family 5. Positive segregation of *JUP* c.475G>T (p.(Val159Leu)) with the pathology in the family. This variant was previously considered as a pathogenic variant (CM1010258). Case II.1 is a 30-year-old man with a positive basal BrS ECG, syncope, and positive EPS. His brother (II.2), a 26-year-old man diagnosed with a positive ECG after flecainide test, and a positive EPS was also a carrier of the detected variation. Both relatives carry an ICD. Family history includes BrS diagnosed in their father (I.1).

**Fig 6:**
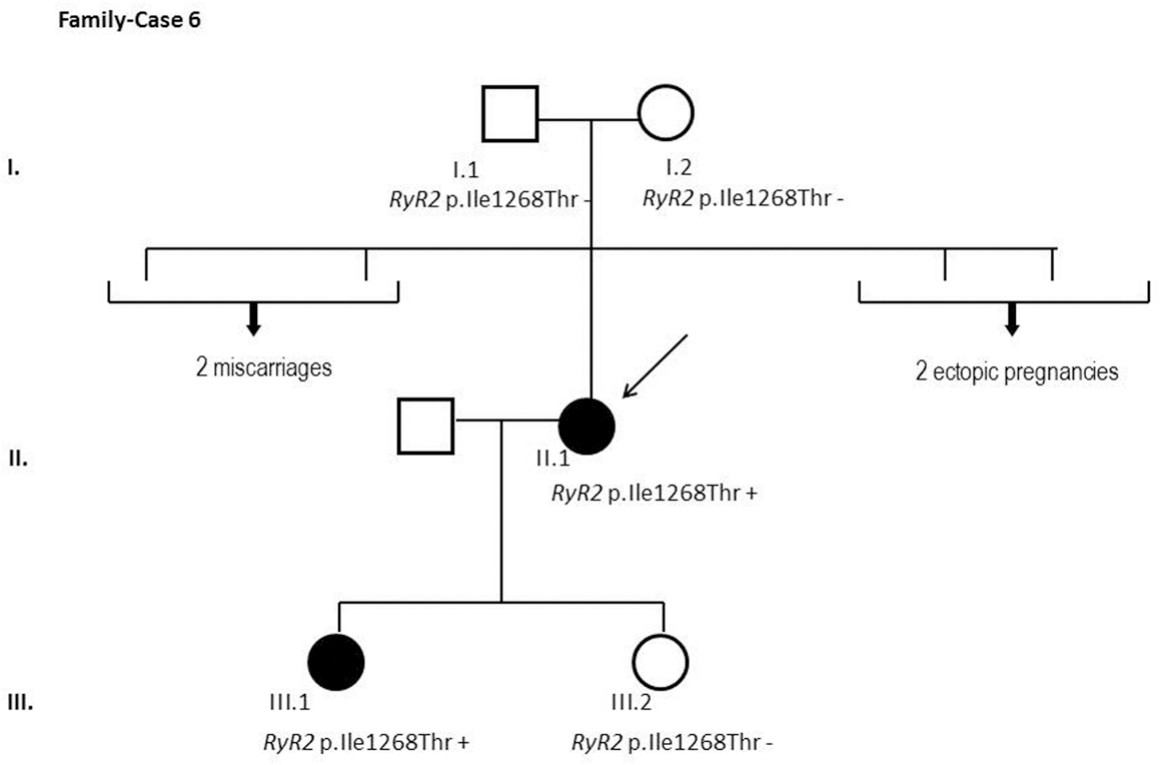
Case-Family 6. Positive segregation of the *de novo* detected variant *RYR2* c.3803T>C (p.(Ile1268Thr)) with the pathology in the family. The index case (II.1) is a 48-year-old woman, previously asymptomatic, with a positive flecainide test and negative EPS study. The variant was also detected in one of her two daughters (III.1), clinically affected of BrS showing a positive ajmaline test. The second daughter (III.2) was clinically unaffected.

**Fig 7:**
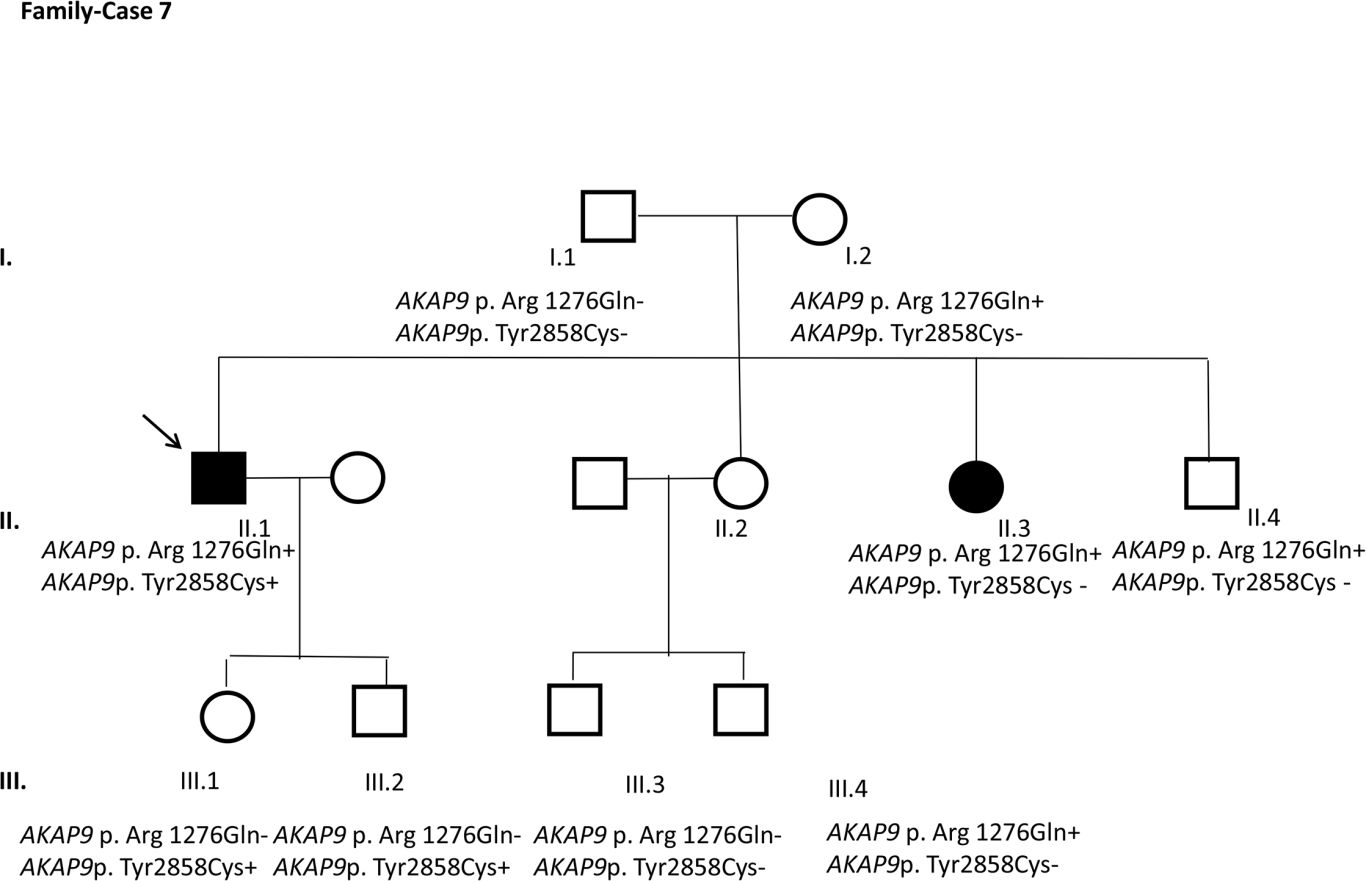
Case-Family 7. Incomplete penetrance pattern of both variations in *AKAP9* c.3827G>A (p.(Arg1276Gln)) (rs146797353) and the *de novo* detected variant *AKAP9* c.8573A>G (p.(Tyr 2858Cys)) in the family. The index case, II.1, is a 44-year-old man with a positive basal ECG, previously asymptomatic, and positive EPS and flecainide test. No family history of SD was reported. The variant *AKAP9* c.3827G>A (p.Arg1276Gln) was also detected in 4 more relatives: his asymptomatic mother (I.2); one clinically-affected sister with a positive flecainide test (II.3); an asymptomatic brother with negative ajmaline test (II.4); and an asymptomatic nephew (III.4). Familial segregation showed that the variant was absent in four clinically unaffected relatives: I.1, III.1, III.2, and III.3. Additionally, the index case showed another *de novo* variant in *AKAP9* c.8573A>G (p.(Tyr2858Cys)), also identified in his two asymptomatic children (III.1 and III.2). This last variant, absent in the remaining available relatives, is also absent in the international databases.

**Fig 8:**
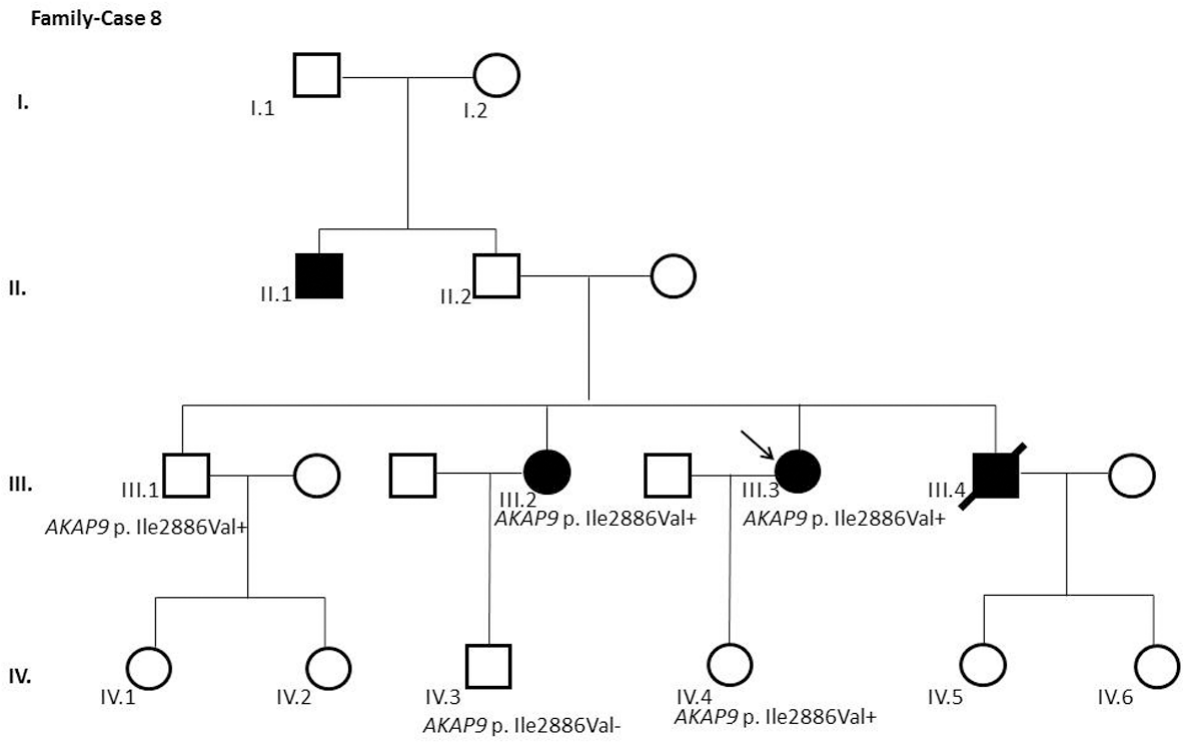
Case Family 8. Incomplete penetrance pattern of *AKAP9* c.8656A>G (p.(Ile2886Val)) (rs143283097) in the family. Family history of sudden death was related. Individual III.3 is a 43-year-old woman previously asymptomatic, with a negative basal ECG but positive procainamide test and EPS. The variant was detected in her asymptomatic daughter (IV.4) and also in (III.2), clinically affected. Her asymptomatic brother (III.1) did carry the variation. The asymptomatic nephew (IV.3) did not carry the variant.

**Fig 9:**
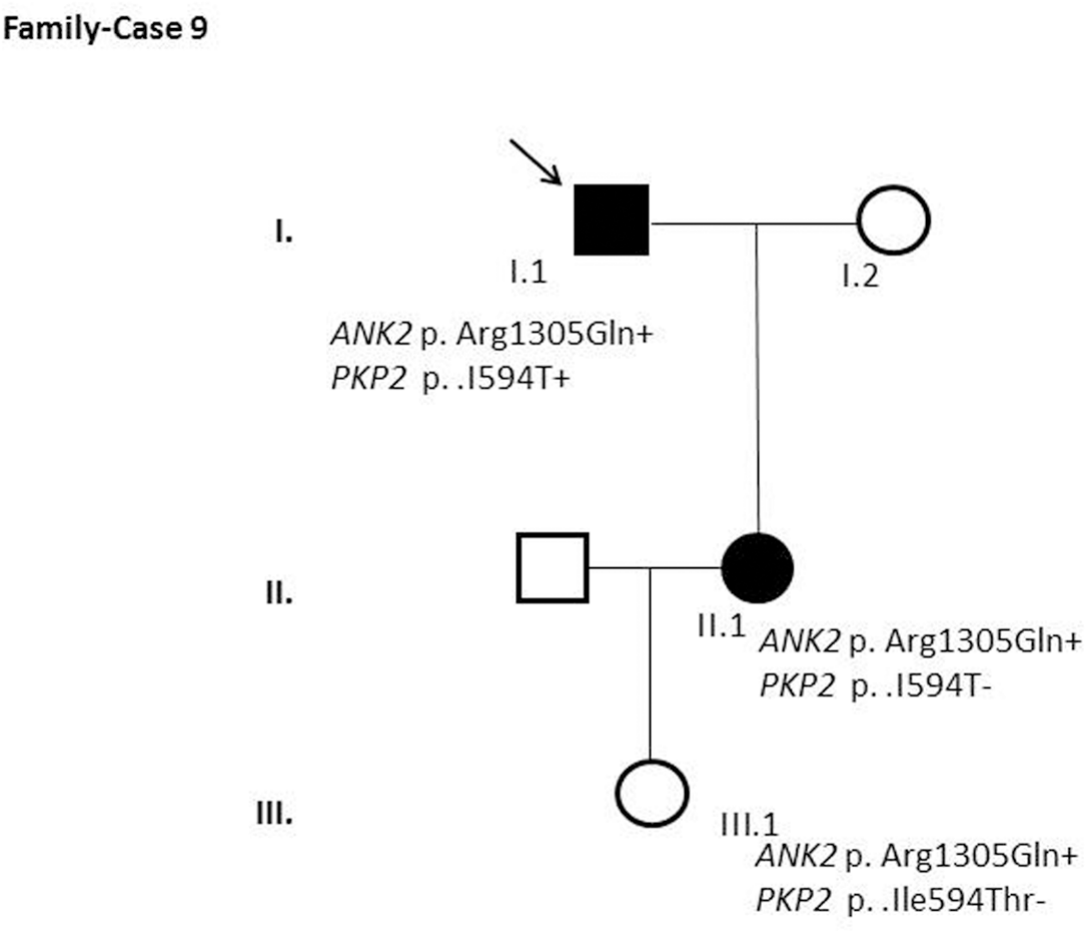
Case-Family 9. Incomplete penetrance pattern of *ANK2* c.3914G>A (p.(Arg1305Gln)) and the lack of penetrance of *PKP2* c.1781T>C (p.(Ile594Thr)) in the family. Individual I.1 is a 74-year-old symptomatic man with both a positive ajmaline test and EPS. His 51-year-old daughter (II.1) was also diagnosed with BrS, showing a positive ajmaline test. Her 21-year-old granddaughter (III.1) is asymptomatic and had a negative ajmaline test. p.Arg1305Gln was not previously described and is predicted deleterious. A second variant considered deleterious, *PKP2* (p.(Ile594Thr)), was detected in the index case (I.1), but was absent in II.1 and III.1.

**Fig 10:**
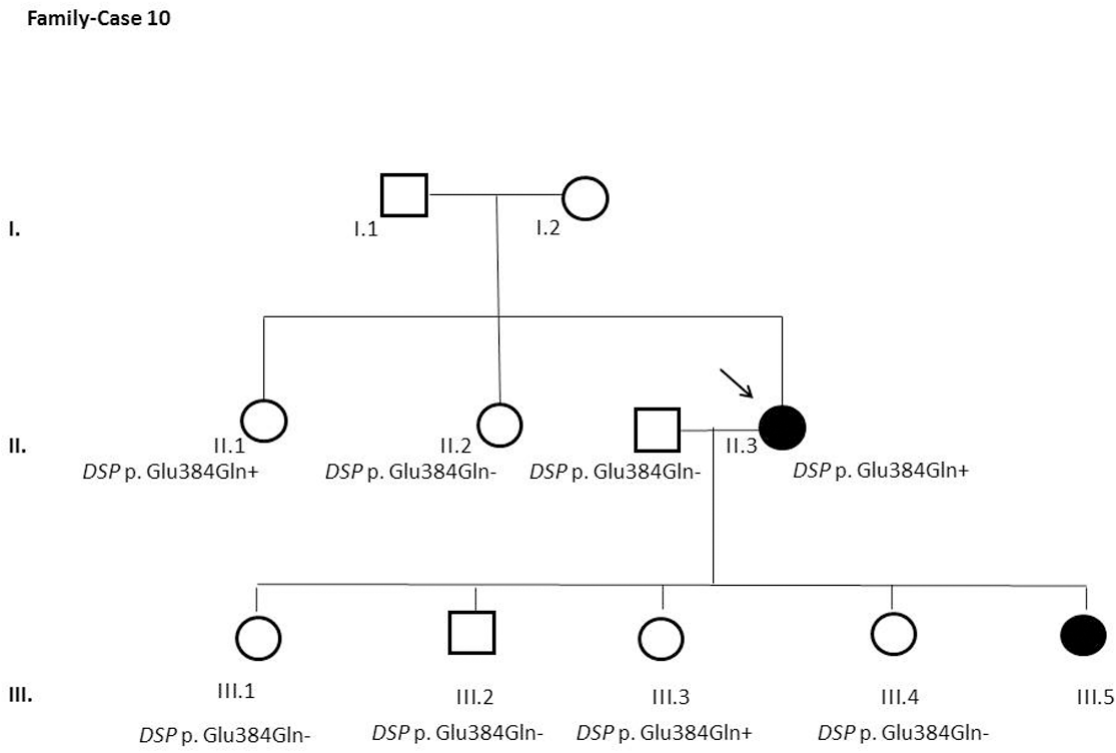
Case-Family 10. Incomplete penetrance of *DSP* c.1150G>C (p.(Glu384Gln)). Individual II.3 is a 48-year-old woman clinically affected with BrS. It was also confirmed in two more relatives: both asymptomatic sister and daughter (II.1 and III.3). The variant was absent in another sister and two daughters and a son (II.2, III.1, III.4 and III.2). Family history includes SCD in a 12-year-old daughter (III.5).

**Fig 11:**
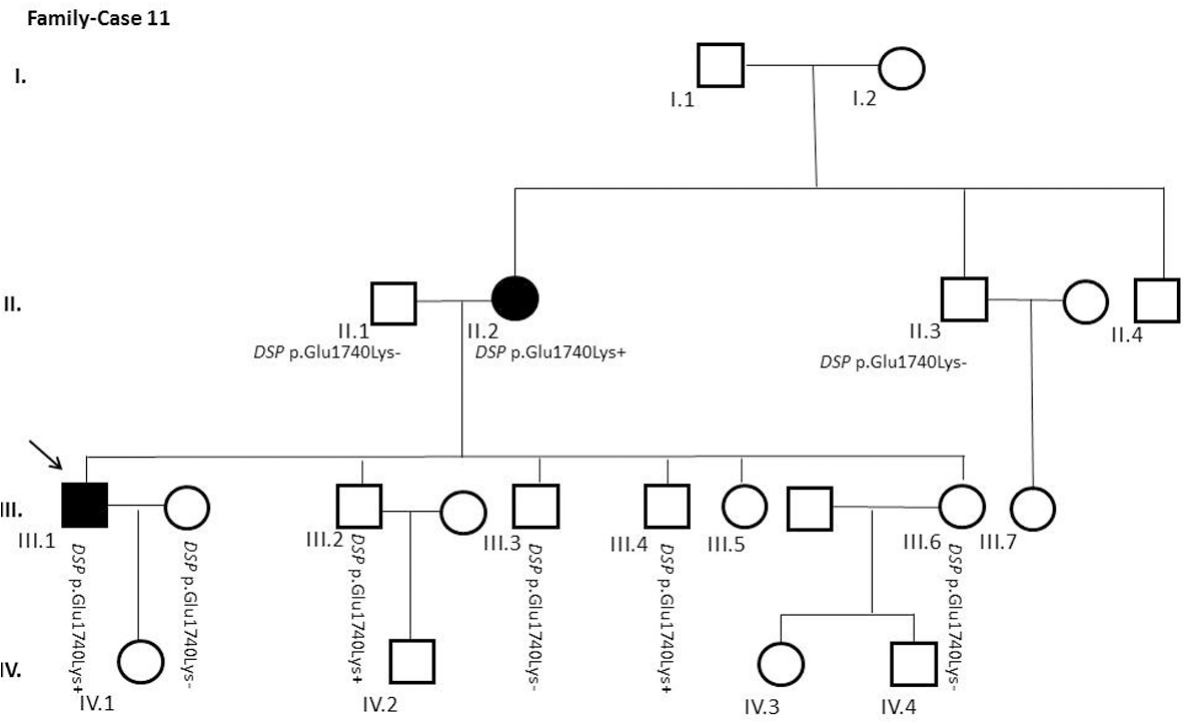
Case-Family 11. Incomplete penetrance of *DSP* c.5218G>A (p.(Glu1740Lys)) (rs142885240). Individual II.2 is a 68-year-old woman, previously asymptomatic but with positive basal ECG and procainamide test. An ICD was implanted. Her affected son (III.1) and two clinically asymptomatic sons (III.2 and III.4) also carry the same genetic variant. Her husband (II.1) and children (III.3 and III.6), also asymptomatic, did not carry the variant.

**Fig 12:**
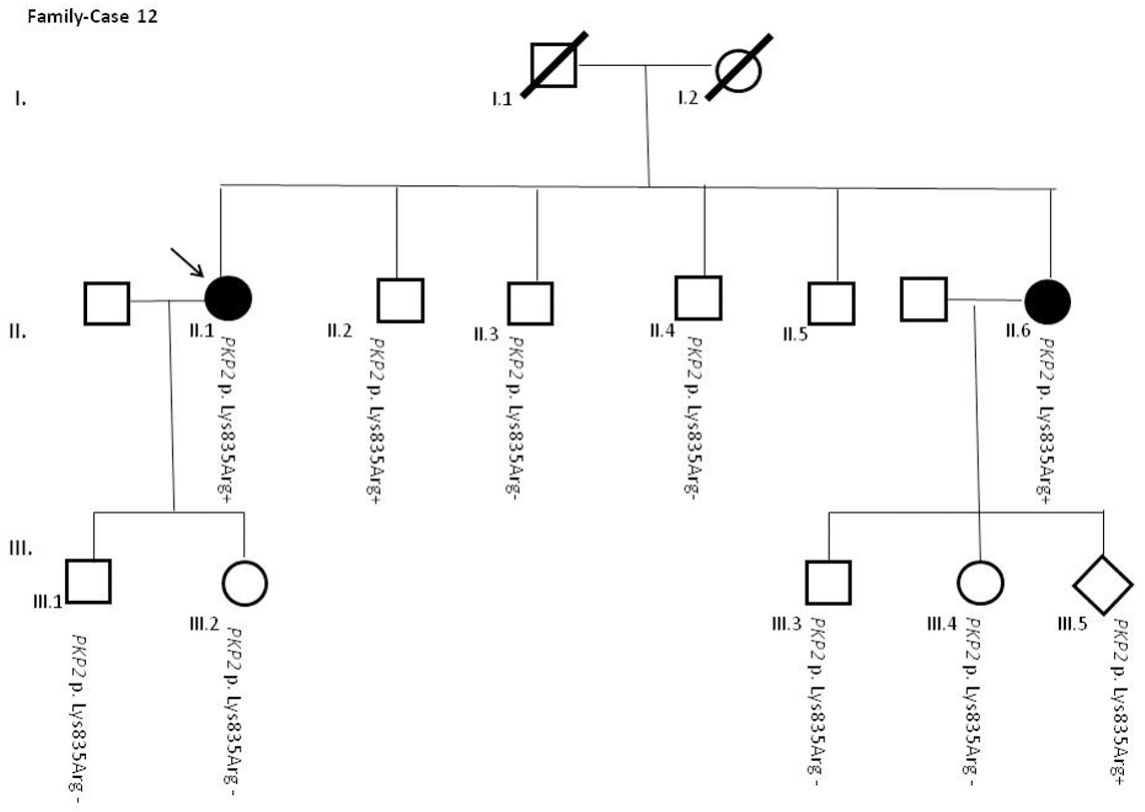
Case-family 12. Incomplete penetrance of *PKP2* c.2504A>G (p.(Lys835Arg)) (rs372729739) in the family. Individual II.1 is a 38-year-old woman who suffered several syncopes and showed positive ECG-Type I after ajmaline test and had no family history of sudden death. Her son and daughter (III.1 and III.2) and two brothers (II.3 and II.4), all asymptomatic, did not carry the genetic variant. The variant was identified in an asymptomatic brother (II.2), and an affected sister (II.6) and child (III.5).

#### Group 1

Positive segregation (see Fig 1 to Fig 12, S4 Table and Table 2 for segregation results, familial clinical history and rare variant report and classification). Incomplete penetrance pattern of the detected variants is also considered into this first group. This group includes 12 cases.

In the first 6 cases, the detected genetic variation showed complete penetrance. In cases 1 and 2 (Figs 1 and 2) the segregation variant was detected in the *ANK2* gene. In case 3, two variations, in *CASQ2* and *KCNJ2*, correlate with the disease in this large family with a history of SCD (Fig 3). In case 4 the variant was detected in *HCN4* (Fig 4). In case 5, the variant was detected in *JUP* (Fig 5). Case 6 showed a novel de novo variation in the *RyR*2 gene (Fig 6). In these 6 cases, 6 additional potentially functional affecting variants were also identified. However, those 6 variations did not segregate with the pathology in the family.

The remaining 6 cases in this first group showed an incomplete penetrance pattern of inheritance (Table 2). In two cases, 7 and 8 (Figs 7 and 8), the genetic variation was detected in *AKAP9*. Case 9 (Fig 9) had two genetic variants, in *ANK2 and PKP2*. The remaining three cases had variants in *DSP* and *PKP2* (Fig 10 to Fig 12).

#### Group 2

Unclear segregation (see Table 2 for rare variant report and classification).

The second group includes those cases in which DNA was not available for genetic resequencing from relatives, therefore prohibiting ascertainment of segregation. This group comprises 5 cases (Table 2), in which 6 variations were detected in 6 different genes.

In case 13 the genetic variation detected in *ANK2* c.5758G>A; (p.(Gly1920Arg)) was previously catalogued as rs140189724 and predicted as neutral. Family history included a brother affected with BrS. Case 14 was a carrier of a previously undescribed genetic variation in *CACNA1C* c.5875G>C; (p.(Gly1959Arg)), predicted as neutral. He was a 33-year-old man diagnosed after an aborted SCD event, with a positive ECG at baseline. His father experienced SCD. Case 15, a 40-year-old man diagnosed with a positive basal ECG, carried the genetic variation in *DSG2* c.3209C>T (p.(Thr1070Met)); rs149617776, which is predicted neutral. Family history included a 30-year-old brother who died suddenly. Case 16, whose father died suddenly, was a 41-year-old man with the *DSP* variant c.8455A>C (p.(Met2819Leu)); rs138329459 CM132698) [12], which is predicted as neutral. Two variations, *RyR2* c.5056C>T (p.(Leu1686Phe)), which is novel, and *PKP2* c.302G>A (p.(Arg101His)); rs149542398, were detected in case 17, a 46-year-old man with a positive ECG at baseline. Both were predicted as deleterious. Family history included two uncles who died suddenly.

#### Group 3

Negative segregation (see Table 2 for rare variant report and classification).

The third group includes 5 cases in which the variations detected did not segregate with the phenotype in the available family members (Table 2); these cases therefore remain undiagnosed genetically. Interestingly, the variants detected were predicted as pathogenic by informatics tools.

In case 18 the variation *ANK2* c.2945G>A (p.(Arg982Gln)), not previously described, was identified and was predicted deleterious. Two of the case’s four children were also diagnosed with BrS but did not carry the detected variant. Case 19 belonged to a large family with history of sudden death. *CACNA1C* c.2449C>T (p.(Pro817Ser)); rs112532048, considered neutral, was detected in 13 relatives. Two affected individuals did not carry the genetic variation. Case 20 carried the variant *CACNB2* c.1925T>C (p.(Ile642Thr)), considered neutral. One of the daughters was affected with BrS but did not carry the genetic variant, thus indicating the variation was not causative of the pathology. Case 21 carried *DSG2* c.1003A>G (p.(Thr335Ala)); rs191564916, considered neutral. He had a positive basal ECG and syncope. His father and one brother showed a positive flecainide test but did not carry the variation. Case 22 carried the genetic variation *DSG2* c.473T>G (p.(Val158Gly)); rs191143292_CM070921, considered deleterious. He was a 38-year-old man diagnosed with BrS after a positive basal ECG. Family history included sudden death of his father and brother, as well as positive flecainide test in another brother, who carried the same genetic variant. His two sons were also diagnosed with BrS, but only one of them carried the variant.

In recent years, some compendiums focusing on the genetics of BrS have been published. These studies performed Sanger sequencing of known BrS-related genes and showed that 20 to 25% of BrS patients carry a pathogenic variation in the *SCN5A* gene. However, nearly 70% of BrS patients remain without genetic cause after genetic analysis. Accordingly, the Canadian Cardiovascular Society/Canadian Heart Rhythm Society expert consensus statement suggested genetic testing only of the *SCN5A* gene for any patient diagnosed with BrS. This consensus recommends that the genetic testing of minor BrS-associated genes should only be considered under special circumstances, given the difficulty of drawing clinical conclusions from few patients with these genetic defects and the low yield of genetic testing in these less prevalent genes [3].

However, the development of high-throughput technologies allows the screening of large sets of genes at once. Therefore, NGS technology is progressively being incorporated into clinical diagnosis. To the best of our knowledge, our study is the first comprehensive genetic analysis using NGS technology to identify genetic variants associated with BrS. Indeed, in our study, NGS resequencing of individuals with BrS detected a total of 30 rare genetic *missense* variants in the heterozygous state; all were validated by conventional Sanger sequencing.

The study had three main purposes: 1. to determine genetic causality in patients with BrS who did not carry a variation in *SCN5A*; 2. to assess the value of NGS technology and bioinformatics tools in determining causality; and 3. to use these data to interpret genetic causality in the event that these were cases of unexplained SCD. These objectives are critical to determining whether there exists a role for NGS in the clinical diagnosis of sudden death, especially considering that the current recommended strategies to determine causality are not definitive: segregation analyses may not always be possible, bioinformatics pathogenicity prediction may not always be accurate, and functional analyses are never performed for clinical determination of causality. The lack of this in vitro/in vivo analysis is the main limitation to determining the score of pathogenicity. However, using NGS data, and accepting present limitations and present knowledge, we have identified several variants potentially causative of BrS, based on low prevalence in the general population.

### ANK2

Previous studies demonstrated the role of ankyrin, especially ankyrin-G, in the sodium channel trafficking that results in several life-threatening conditions including cardiac arrhythmias. While ankyrin-G is required for the targeting of Nav1.5 to the cardiomyocyte intercalated disc, ankyrin-B is required for targeting the Na^+^/Ca^++^ exchanger and Na^+^/K^+^ ATPase to transverse tubule membranes in the heart. Interestingly, ankyrin-B knockout mice exhibit a delayed opening of the sodium channel, although no evidence of an interaction between the sodium channel and ankyrin-B has been reported so far. *ANK2* (encoding ankyrin-B) was previously associated with LQTS type 4, producing a loss-of-function effect. A total of 5 genetic variations in *ANK2* were detected in 4 cases. Cases 1 and 2 had complete penetrance; in case 13 a segregation study could not be completed as DNA was not available from relatives; and in case 18 the variant did not segregate with the pathology in the family. The incidence of these rare variants implicates *ANK2* as a potential candidate gene in BrS. Further functional and molecular studies of the detected variants should clarify the molecular mechanisms related to pathogenic variations in the *ANK2* gene that underlies a BrS phenotype [13–15].

### CASQ2

Homozygous variations in *CASQ2* are associated with recessive forms of catecholaminergic polymorphic ventricular tachycardia (CPVT), an inherited arrhythmogenic disorder associated with SCD. However, *CASQ2* was not previously associated with BrS. *CASQ2* encodes calsequestrin, which is located inside the sarcoplasmic reticulum (SR) and buffers the physiological intra-SR calcium. In case 3, the genetic variant in *CASQ2* was accompanied by a second variant, in *KCNJ2*, which also segregated with the pathology. Variations in *KCNJ2* are associated with LQTS type 7 and short QT syndrome. A related gene, *KCNJ8*, has been associated with BrS, though at present it appears that some of the variations are actually normal variants in the general population. In addition, in this family, two members carry both genetic variations are also clinically diagnosed for AF. This fact agrees with the pathogenic variations reported in the *KCNJ2* gene in patients diagnosed with AF [16],[17].

### HCN4

*HCN4* encodes the potassium/sodium hyperpolarization-activated cyclic nucleotide-gated channel 4 and was previously associated with BrS. In the Human Gene Variation Database (HGMD), 10 variations in *HCN4* have been described, with just one of them associated with BrS [18]. The variant we detected was previously reported but not classified for its pathogenic effect. *In silico* database analysis predicted the variation rs200507617 as deleterious.

### Desmosomal proteins

Although, historically, genes encoding desmosomal proteins have not been associated with BrS, Cerrone *et al*. recently defined the co-existence of clinical BrS and genetic variations in *PKP2*, which encodes the desmosomal protein plakophilin [10]. Desmosomal proteins, particularly plakophilin and desmoglein, interact with Nav1.5, [19]. Indeed, a recent review proposed that alterations in the “connexome” are the origin of BrS [20]. This interaction could be a possible explanation of the observed variation in genes encoding desmosomal proteins in BrS in our study. We identified several variations in *JUP*, *DSP*, *PKP2*, and *DSG2*. Variants of these desmosomal proteins are associated with ARVC and dilated cardiomyopathy (DCM), both structural heart pathologies associated with SCD. In case 5 we detected the variant *JUP* p. Val159Leu, previously associated with ARVC (CM1010258) [21]. Cases 21 and 22 carried a genetic variant in *DSG2*. Although these did not segregate with the pathology, one of the variations, rs191143292, was already published as pathogenic and causal for ARVC [22]. These genetic variations detected in our cohort should be further studied to clarify their role in BrS.

### RYR2

Genetic variants in the *RyR2* gene were also detected in our cohort. *RyR2* encodes the ryanodine receptor 2. Variations in this gene have been associated with CPVT and LQTS. Arrhythmia susceptibility in LQTS individuals may be due to two distinct Ca^2+^ mechanisms: the early afterdepolarizations (EADs), which are driven by reactivation of the L-type calcium channels during repolarization, and a mechanism that may occur during diastole (delayed afterdepolarizations, DAD) via Ca^2+^ cycling by altering the function of the cardiac RyR2 channel [23]. The detected variation segregates in case 6, but in case 17 segregation could not be established. To date, no association between BrS and this gene has been described, though calcium channels have been associated with the disease [24].

### Calcium channels

Several genetic variations in two calcium channel genes were identified in our samples. Two variants were detected in *CACNA1C*, which encodes Cav 1.2. However, segregation analysis was not available in case 14, and no segregation was shown in case 19. Similarly, the segregation study was negative in case 20 for the genetic variant in *CACNB2*. Although both genes have been associated with BrS [24], analysis of variants did not clarify their pathogenic role in BrS. Although calcium channels and their associated proteins have been proposed as candidates for BrS pathogenesis, we cannot make any conclusion for their role following NGS and segregation analysis of the detected variants.

### AKAP9

Two cases showed genetic variations in *AKAP9*, both with incomplete penetrance. *AKAP9* encodes Yotiao protein, associated with LQTS and having a critical contribution to I_ks_ regulation [25]. As other potassium channels, such Kv4.3, MiRP1, and MiRP2, have been associated with BrS, similar mechanisms could underlie BrS in these cases.

### De novo variants in *RyR2 and AKAP9*

Two de novo variations were identified; one in case 6, *RyR2* p.I1268T/c.3803T>C, and one in case 7, *AKAP9* (p.(Tyr2858Cys)). The latter was detected together with *AKAP9* (p.(Arg1276Gln)). However, in case 7 this novel variation did not correlate with the pathology, as the affected individual II.3 (Fig 8) did not carry the variation.

Only 3 of the 12 genes in which we have identified potentially pathogenic variations were previously associated with BrS—four, considering the recently published data in *PKP2* [10]. All these genes are likely new candidate genes as all are already associated to arrhythmogenesis and SCD. In addition, the *in silico* predictor tools together with the genetic variation frequency in general population and, most importantly the segregation analysis in relatives make all these genes, make these genes good candidate genes to further investigate their role in the genetic substrate of BrS. These results need to be further evaluated in larger BrS cohorts to investigate their function in BrS pathogenesis. The identified variants should be examined in cellular models to further confirm causality. The studies could provide novel mechanistic insights into BrS and arrhythmogenesis.

With these data, we show that several other genes may potentially be causative of the disease, genes that had been previously associated with other arrhythmogenic diseases. As is well known in these other arrhythmogenic diseases, most of the detected variants showed incomplete penetrance. The lack of phenotype in carriers does not preclude segregation, as there can be latent phenotypes, differences in gender and age, or the presence of genetic modulators. In addition, several variants may be identified in these cases. The possible combination of multiple variants could have an essential role in the variability in phenotype expression. Several modifying genetic factors have been described in BrS, and some additional ones have been proposed [26] [27]. Bezzina *et al*. described genetic variants that show a cumulative large effect on BrS susceptibility [28]. As in other SCD-related syndromes, like LQTS, variation in the expressivity related to several genetic variations has been described [29] [30].

Segregation, or lack thereof, was believed to provide a definite lack of association with causality. However, in recent years some data have questioned this for BrS. In some of the families, clinically affected family members do not share the variation in *SCN5A*. Lack of segregation may indicate either that this is not a causative variation or that it is a predisposing genetic factor, albeit weaker than once thought. Previous data show the lack of complete segregation of *SCN5A* variations in some families [31], as well as the presence of induced ST elevation as the first ECG alteration just before the SCD event [32]. This raises the hypothesis that the BrS ECG could actually be an electrical pattern triggered by a genetic predisposition to electrical instability, but which goes beyond pure Mendelian genetics. It is in this context that the data presented here can be scientifically understood. It is unlikely that there is an additional presence of variations associated with other diseases in patients with BrS, given that these are such rare diseases. The ECG pattern could be the result of combining either a strong genetic background (variation in *SCN5A*), or several minimal genetic alterations, with a certain structural makeup of the right ventricle that predisposes to slowing of conduction.

Several new bioinformatics tools enable an approach to the level of pathogenicity, which assesses, among other factors, the absence of the variation in the normal population as well as the effect of the variation in the protein. The localization of a rare *missense* variant in a critical area of the cardiac ion channel protein, or a truncating variation in major associated genes, are supposed to be pathogenic [33]. *In silico* prediction tools are thought to be useful when they show a deleterious score, but its value in neutral and benign scores seems less robust. In cases included in group 3, Segregation analyses definitively indicated that the variants were not causative, but it remains to be seen if, in the future, they will be classified as genetic modulators of the phenotype.

New minority genes had been recently associated with BrS (*SCN2B*, *SCN10A*, *RANGRF*, *SLMAP*, *ABCC9*, *KCNJ8*, *KCND3*, *KCNE5*, *CACNA2D1* and *TRPM4*). These genes were not included when the resequencing panel was designed. Although the percentage of genetically positive cases in those genes is low, these genes should be considered when re-designing resequencing panel for genetic screening of BrS to get more data in several cohorts that support the association of these genes with the disease. Of note, current recommendations on general guidelines are still restricted to *SCN5A*. We can therefore hypothesize that in the clinical arena whole exome sequencing in BrS patients could result even in more harm at the moment due to the large amount of data generated and needed to be interpreted. However, in large well-characterized families, the whole exome sequencing should be considered for research purposes.

An exhaustive bioinformatic analysis did not detect insertions/deletions or copy number variations in our samples. This analysis was developed by using an in-house bioinformatics pipeline (manuscript under construction).

## Conclusions

In conclusion, this work highlights the challenges that we face in the diagnosis of unexplained SCD. We have used BrS as a paradigm of unexplained sudden death in the structurally normal heart. We show that the identification of a genetic defect in the victim is not equivalent to providing a clear diagnosis. The identified genetic defects have provided a potential diagnosis of BrS, LQTS, CPVT, and ARVC. Massively parallel sequencing confirms that it is not always possible to determine the phenotype from genetic data. The inclusion of functional analyses could be determinant to assign a definite diagnosis, but this is not possible at the present rate of variation detection. Therefore, the introduction of NGS technology has led to more questions unanswered compared with the pre-NGS era. This was an expected challenge that always comes about in these approaches when large lists of genes are interrogated. The sequencing process of those genes gives huge amounts of data that had to be stored, analyzed and biologically and clinically interpreted. Genetic diagnosis by means of NGS is still a challenge since its application to clinical diagnosis requires first an international effort for better understanding of the significance of the rare genetic variations detected. One of the main objectives of the present work is to evaluate the applicability of NGS to clinical (and forensic) diagnosis. After the analysis of our results, this objective appears difficult to assess and only possible from a familiar perspective. However, even with the availability of relatives to enlarge the segregation study, the genetic diagnosis is still difficult to estimate. Detection of novel, presumed disease causing, rare genetic variants in several genes that were not previously associated to BrS are logical consequence of the study. Association of those genes to the disease need to be further evaluated by means of functional studies and replicated in different cohorts before being considered as genetically conclusive for clinical diagnosis. Several reason support that the genes described above should be considered candidate genes for research purposes for better understanding of genetics mechanisms underlying. These reason iclude the role of the proteins encoded by these genes, the low frequency or their absence of the genetic variations in general populations, their potential pathogenicity evaluated *in silico* and when possible, with the segregation analysis performed. These facts suggest a pathogenic role of the genetic variation detected. However, they are considered as VUS after applying the algorithm, mainly due to the lack of functional studies and genotype-phenotype analysis in different familiar cohorts. It is common that rare variants remains as VUS. This observation agrees with the characteristic of pathologies with incomplete penetrance and variable expressivity observed. To ascertain the pathogenic role of the detected variations, genotype-phenotype correlations are required in other familiar cohorts as part of international efforts to further increase understanding of the mutation spectrum underlying BrS. In this direction, GWAS analysis (ya existen) from international consortiums including several BrS cohorts had been published and also are nowadays being developed. These results would result clarifying and open new research perspectives to be evaluated,

Technological progress has been more rapid than our capacity to apply it in the clinical arena. The genetic architecture in terms of genes involved, rare and common genetic variants, and modifying factors creates a complex decision network that can only be unraveled by careful clinical and genetic interpretation in a family context. This reinforces the need for accurate and thorough familial investigation, but also the importance of having the data handled by experts in the field, so as to understand the value but also the limitations of these NGS tests.

## Supporting Information

S1 Fig Sequencing statistics. Percentage of base pairs covered at a given sequence depth across all samples (Figure A). Average Mapping Quality in phred-score scale for all filtered reads by sample (mean mapping quality of 35,68 ± 3,10 ranging from 30,48 to 40,13) (Figure B). Evenness of coverage for all samples. Black bars indicates the target base pair coverage per sample by at least 1 read (mean coverage of 97,83 ± 0,36% ranging from 96,40% to 99,24%) whereas white bars indicates the target base pair coverage per sample by at least 20 reads (mean coverage of 96,06% ranging from 94,45% to 96,97%). The green dashed line represents the mean of coverage 1x across all samples (SD = 0,43) while the green solid line indicates the mean of coverage 20x across all samples (SD = 0,51) (Figure C).
(TIFF)

S2 Fig ECG of a negative case after NGS analysis. (TIFF)

S3 Fig ECG of an index case. (TIFF)

S1 Methods (DOC)

S1 Table NGS Run Statistics and Target Coverage per sample. (DOC)

S2 Table NGS Run Statistics and Target Coverage per batch. (DOC)

S3 Table Consistently low-covered regions. Captured regions with less than 95% of their sequence covered at 20x (considered if detected in at least 5 samples). Chromosomic Region: coordinates of the region (hg19/GRCh37); Gene: HGNC gene symbol; Ensembl isoform, corresponding Ensembl gene isoform; Exon num, corresponding exon number; Num.samples, amount of samples in which region is tagged.
(DOC)

S4 Table Clinical and familiar information of rare genetic variant carriers identified. *Yes: Aborted Sudden Cardiac Death or syncope of suspected cardiac origin. A: American, EU:European; ICD: Implantable Cardioverter Defibrillator. FM: Family Members; GCR: Genetic Carrier Relatives; NGC No genetic carriers relatives.
(DOC)
