# Supplementary figures and images for: Genetic Analysis of Arrhythmogenic Diseases in the Era of NGS: The Complexity of Clinical Decision-Making in Brugada Syndrome
Source: PLoS One. 2015 Jul 31;10(7):e0133037. doi: 10.1371/journal.pone.0133037 (PMC4521779; doi:10.1371/journal.pone.0133037)

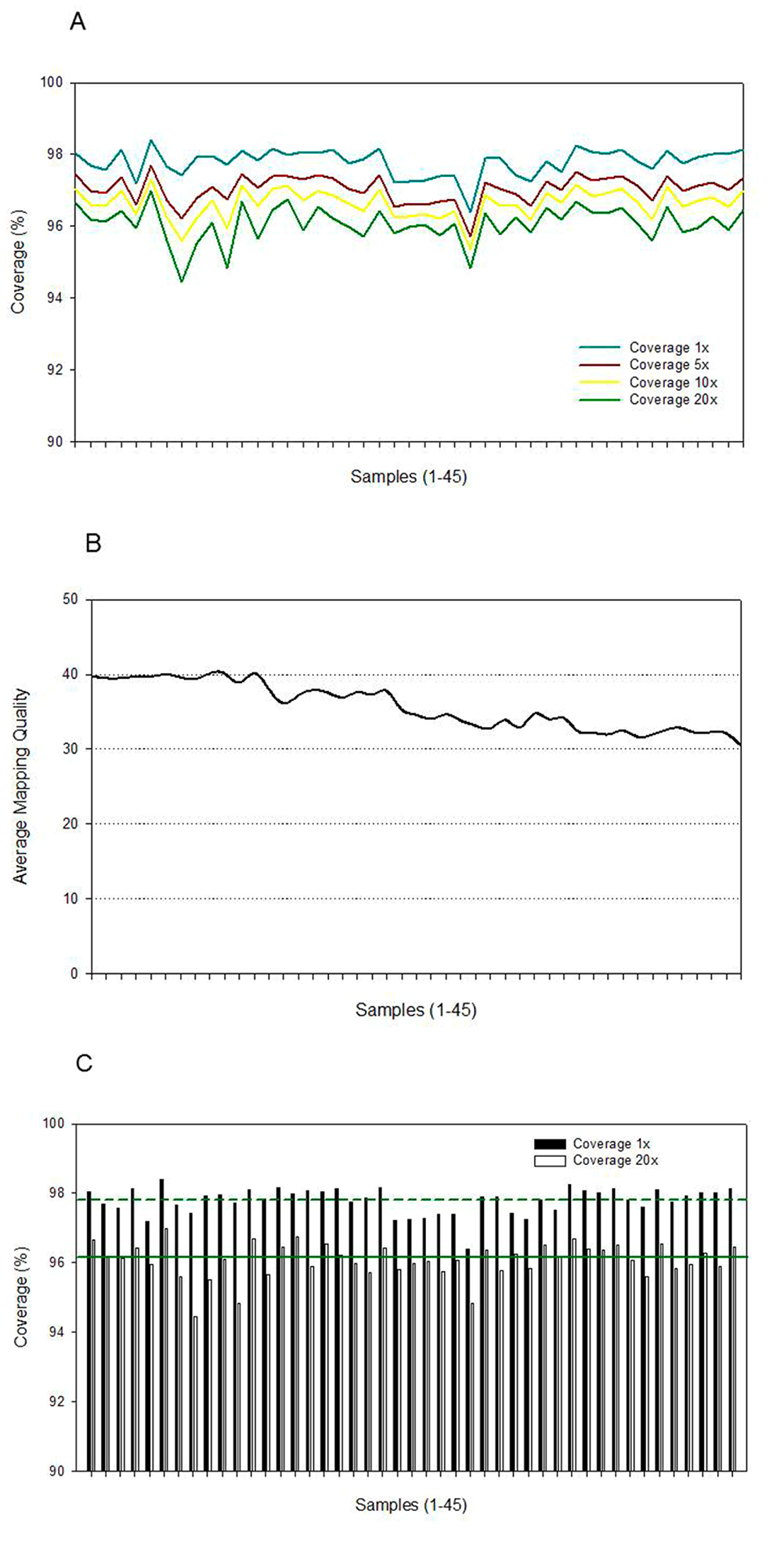

Supplement: S1 Fig — Percentage of base pairs covered at a given sequence depth across all samples (Figure A). Average Mapping Quality in phred-score scale for all filtered reads by sample (mean mapping quality of 35,68 ± 3,10 ranging from 30,48 to 40,13) (Figure B). Evenness of coverage for all samples. Black bars indicates the target base pair coverage per sample by at least 1 read (mean coverage of 97,83 ± 0,36% ranging from 96,40% to 99,24%) whereas white bars indicates the target base pair coverage per sample by at least 20 reads (mean coverage of 96,06% ranging from 94,45% to 96,97%). The green dashed line represents the mean of coverage 1x across all samples (SD = 0,43) while the green solid line indicates the mean of coverage 20x across all samples (SD = 0,51) (Figure C). (TIFF) [file pone.0133037.s001.tiff]

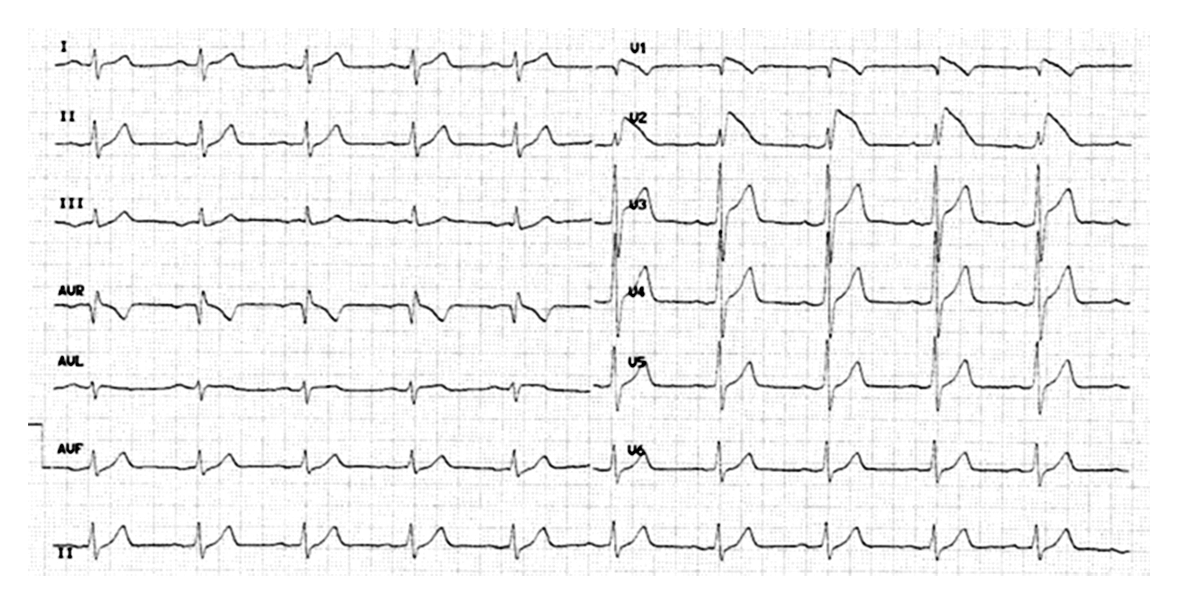

Supplement: S2 Fig — (TIFF) [file pone.0133037.s002.tiff]

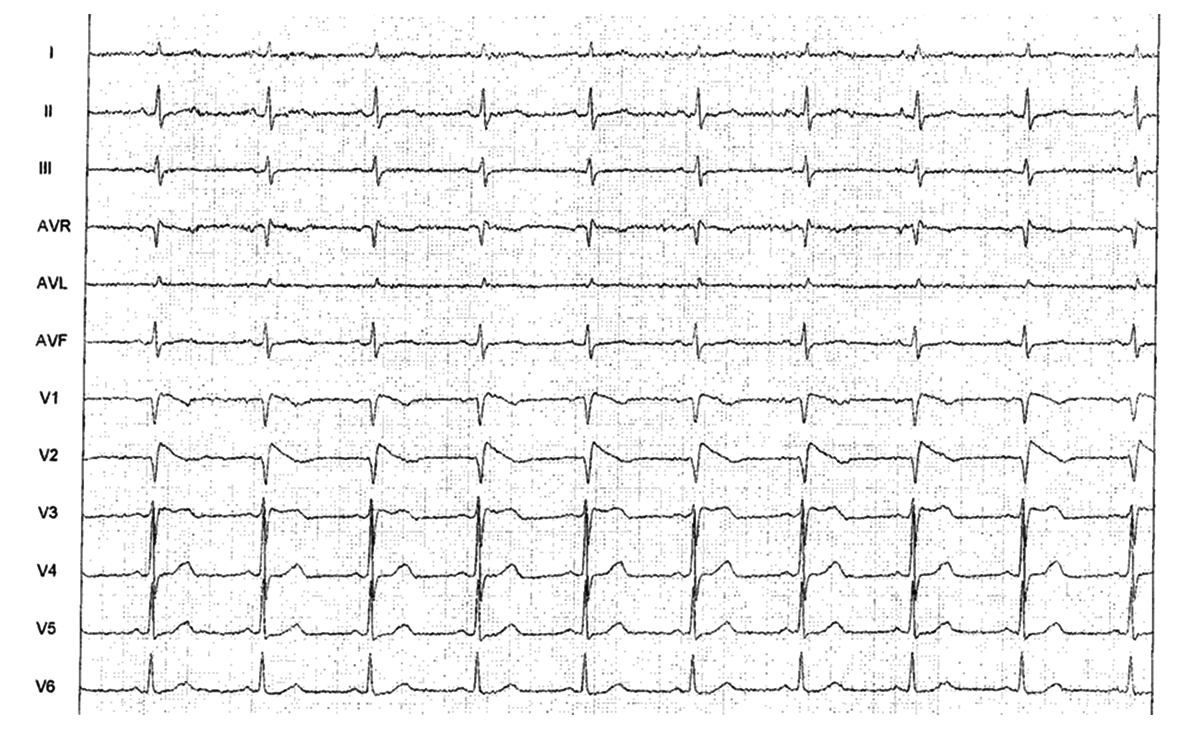

Supplement: S3 Fig — (TIFF) [file pone.0133037.s003.tiff]
