# Supplementary material for: Genetic Analysis of Arrhythmogenic Diseases in the Era of NGS: The Complexity of Clinical Decision-Making in Brugada Syndrome
Source: PLoS One. 2015 Jul 31;10(7):e0133037. doi: 10.1371/journal.pone.0133037 (PMC4521779; doi:10.1371/journal.pone.0133037)
Supplement: S1 Methods — (DOC) [file pone.0133037.s004.doc]

**SUPPLEMENTAL MATERIAL**

**Supplemental Methods**

**DNA sample preparation**

From each BrS patient clinically evaluated, genomic DNA was extracted with Chemagic MSM I from whole blood (Chemagic human blood) or saliva (Chemagic Oragene Saliva) and amplified by polymerase chain reaction (PCR) using intronic primers for each 27 codifying *SCN5A* exons ([RefSeq: NM_198056](http://www.ncbi.nlm.nih.gov/nuccore/NM_198056)) (NCBI -National Center for Biotechnology Information-, http://www.ncbi.nlm.nih.gov/). The PCR product was purified by ExoSAP-IT and directly sequenced by dideoxy chain-termination method in ABI Prism Big Dye® Terminator v3.1 Cycle Sequencing Kit (Applied Biosystems, USA). Sequencing process was processed in a 3130xl Genetic Analyzer, Applied Biosystems, and analyzed by means of the SeqScape Software v2.5 (Life Technologies) comparing obtained results with the reference sequence from hg19/GRCh37. Qubit Fluorometer (Invitrogen) and Nanodrop 1000 (Thermo Scientific) were applied to asses DNA quantity and quality before NGS. To asses DNA integrity, high molecular weight DNA was evaluated by image 0.8% agarose gel with high molecular weight marker. For selecting the index case in each family, DNA quality control was conducted in terms of integrity, quantity and purity, as crucial points to begin NGS approaches. A total of 3µg of not degraded DNA was dissolved in 100 µL of water and fragmented with Covaris AFA system (Covaris Inc; Woburn, MA) to a size of 100-300 bp, checked in Bioanalyzer 2100 (Agilent Technologies).

**Targeted Enrichment Resequencing Panel**

Although all the samples included were negative after conventional Sanger sequencing of *SCN5A*, this gene in the NGS designed panel allows an internal control of the resulting genotypes.

Current literature was accessed and genes of interest were selected and included in the custom panel by means of free access databases (http://www.ensembl.org/index.html; http://genome.ucsc.edu/).. The resequencing panel was designed by using eArray program (Agilent Technologies). DNA sample preparation and the target enrichment resequencing panel protocols are shown in supplemental material. Quality control of the NGS strategy: Target enrichment and Run statistics are shown in the supplemental tables 1 and 2. Consistently low-covered regions are shown in Supplemental table S3.

The 28 selected genes (191.12 Kb) in the 45 DNA samples, were enriched using SureSelect Custom Target Enrichment System kit (Agilent Technologies) following manufacturing protocol "SureSelect Target Enrichment System for SOLiD Fragment and Paired-End Sequencing (version 1.3)". Barcoded libraries were pooled in 4 pools, and a mini-scale emulsion PCR of each pool was performed following manufacturing protocol "Applied Biosystems SOLiD^TM^ 4 System Templated Bead Preparation Guide (4448378 Rev.B)". A paired end sequencing approach were performed. Sequenced reads were about 50 and 35 bp and were mapped using Bioscope v1.3.1. Duplicated reads were filtered with Picard 1.68. Variants were identified using Bioscope v1.3.1 and GATK v1.5. Annotation of the variants was performed using ANNOVAR 2012Apr17.

The identified variants were subsequently filtered by their allelic frequency considering rare variants those with a MAF under 1% in Exome Variant Server and 1000 genomes databases, variants absent in dbSNP130 and those in which the percentage of mutated allele was detected over 15% of the reads. Despite the effort of validating all the variants in these range (15% of mutated allele detection), allowing a large false positive rate, it was projected to minimize potential sources of false negatives. After those filter, each variant was further evaluated by means of Integrative Genomic Viewer (IGV) to visualize the genomic data obtained together with genomic annotations . Previous data from conventional Sanger sequencing of *SCN5A* was compared to get a false negative ratio of the common variants identified.

***In Silico* Analysis of Rare Nonsynonymous Variants**

The Consensus Deleteriousness Score (ConDel) approach is based on a weighted average of normalized scores of ﬁve predictive tools: Log R Pfam E-value (Logre), MAPP Mutation Assessor (Massessor) Polyphen2 (PPH2) and SIFT. Condel integrates the output of these methods and their related computational tools into a unified classification to categorize *missense* Single Nucleotide Variants (SNVs) as probably deleterious or probably neutral polymorphisms. Taken together, the internal scores of the five predictive tools, although different in nature, reflect the probability that an amino acid change will be accepted at a given position of a protein sequence. After consulting, several factors are evaluated: is calculated a posteriori probability that a mutation is deleterious; a score for a given amino acid and if it does represent the violation of physic-chemical constraints at a position of the protein sequence; the putative alteration in the fit of the protein domain where the mutation is located; and also scores of the amino acid substitutions on the basis of the conservation of residues that define subfamilies within protein families in a multiple-sequence alignment . Together with Condel, also Mutation Taster (http://www.mutationtaster.org/), Polyphen (http://genetics.bwh.harvard.edu/pph2/) and Provean (http://provean.jcvi.org/)were applied. Also our published pathogenicity score was assessed (table 3 on the main document).

However, *in silico* prediction of pathogenicity of all the genetic variants detected, especially the novel ones should be interpreted as a probabilistic approach. Genetic variants detected were considered rare variants when the frequency of the minor allele was under 1% (MAF<0,01) in general population after interrogating several locus specific databases that includes dbSNP . Ensembl genome browser and the data of the 1000 Genomes project and EVS (Exome Variant Server, NHLBI GO Exome Sequencing Project _ESP). The Human Gene Mutation Database (HGMD) was also consulted .

**Supplemental Results**

**Quality control of NGS strategy: Target enrichment and Run statistics**

Data from four different batches (ranging from 10 to 12 samples per batch) is shown in supplemental data. The data shown includes: Batch, batch identifier; Total Reads; the average per sample of total reads generated by ABI SOLiD v4.0 TM System (including unmapped reads); Reads On Target, number of reads mapped on regions of interest; Enrichment Fold, how many fold more the regions of interest have been enriched in comparison by other regions; Coverage, base pairs covered at least by 1, 5, 10 or 20 reads, respectively; Average Depth of Coverage, for all samples included per batch; Filtered Reads, reads that have passed the best practices recommended quality control pipeline from GATK software package; Properly Paired Reads, read pairs which both mates map to the same chromosome, oriented towards each other and with an adequate insert size; Average Mapping Quality, in phred-score scale for all filtered reads by sample.

See Supplemental data Table S1: NGS Run Statistics and Target Coverage per sample and Table S2. NGS Run Statistics and Target Coverage per batch.

Each sequencing run produced an estimated average of 6,5x10^6^ 50 and 35-bp, in forward and reverse respectively, long reads, corresponding to an approximate amount of 7x109 bases of total sequence output in the set of the experiment. Next, analyzing the target regions of interest (ROI), nearly 46% of the total reads fell within these regions, and 25% (ranging from 15’3% to 37’3%) of these passed the GATK’s processing quality control pipeline (http://www.broadinstitute.org/gatk/guide/topic?name=best-practices). As a result we obtained a mean number of 684450 properly paired reads (read pairs which both mates map to the same chromosome, oriented towards each other and with an adequate insert size) mapped to the target ROI’s per sample. The average mapping quality of these reads across all samples (in phred quality scale) was always higher than 30 (35,68 ± 3,10) suggesting an appropriate base call accuracy higher than 99,9% (see S1B Fig. ).

Mean depth of coverage over all samples was 620x (ranging from 248 to 951; SD=162,75). The mean target coverage for one or more reads (1x) was 97,83 ± 0,36% and comparing with the coverage for twenty or more reads (20x), that was 96,09 ± 0,51%. To achieve a better visualization on how the percentage of covered base pairs was distributed along different levels of coverage across all samples see S1A S1C Figs. and Supplemental table S3.

**Supplemental References**

1. Gonzalez-Perez A, Lopez-Bigas N. Improving the assessment of the outcome of nonsynonymous SNVs with a consensus deleteriousness score, Condel. American journal of human genetics. 2011;88(4):440-9. Epub 2011/04/05. doi: 10.1016/j.ajhg.2011.03.004. PubMed PMID: 21457909; PubMed Central PMCID: PMC3071923.

2. Sherry ST, Ward MH, Kholodov M, Baker J, Phan L, Smigielski EM, et al. dbSNP: the NCBI database of genetic variation. Nucleic acids research. 2001;29(1):308-11. Epub 2000/01/11. PubMed PMID: 11125122; PubMed Central PMCID: PMC29783.

3. Kersey PJ, Staines DM, Lawson D, Kulesha E, Derwent P, Humphrey JC, et al. Ensembl Genomes: an integrative resource for genome-scale data from non-vertebrate species. Nucleic acids research. 2012;40(Database issue):D91-7. Epub 2011/11/10. doi: 10.1093/nar/gkr895. PubMed PMID: 22067447; PubMed Central PMCID: PMC3245118.

4. A map of human genome variation from population-scale sequencing. Nature. 2010;467(7319):1061-73. Epub 2010/10/29. doi: 10.1038/nature09534. PubMed PMID: 20981092; PubMed Central PMCID: PMC3042601.

5. Stenson PD, Ball EV, Mort M, Phillips AD, Shiel JA, Thomas NS, et al. Human Gene Mutation Database (HGMD): 2003 update. Human mutation. 2003;21(6):577-81. Epub 2003/05/20. doi: 10.1002/humu.10212. PubMed PMID: 12754702.

**Supplemental Tables**

**Supplemental table S1. NGS Run Statistics and Target Coverage per sample.**

**Supplemental table S2. NGS Run Statistics and Target Coverage per batch.**

**Supplemental table S3: Consistently low-covered regions.** Captured regions with less than 95% of their sequence covered at 20x (considered if detected in at least 5 samples). Chromosomic Region: coordinates of the region (hg19/GRCh37); Gene: HGNC gene symbol; Ensembl isoform, corresponding Ensembl gene isoform; Exon num, corresponding exon number; Num.samples, amount of samples in which region is tagged.

**Supplemental table S4_Clinical and familiar information of rare genetic variant carriers identified.** ***Yes: Aborted Sudden Cardiac Death or syncope of suspected cardiac origin.** A: American, EU:European; ICD: Implantable Cardioverter Defibrillator. FM: Family Members; GCR: Genetic Carrier Relatives; NGC No genetic carriers relatives.

**Supplemental Figures**

**S1 Fig.** Sequencing statistics. **S1AFig.** Percentage of base pairs covered at a given sequence depth across all samples. **S1B Fig.** Average Mapping Quality in phred-score scale for all filtered reads by sample (mean mapping quality of 35,68 ± 3,10 ranging from 30,48 to 40,13). **S1C Fig.** Evenness of coverage for all samples. Black bars indicates the target base pair coverage per sample by at least 1 read (mean coverage of 97,83 ± 0,36% ranging from 96,40% to 99,24%) whereas white bars indicates the target base pair coverage per sample by at least 20 reads (mean coverage of 96,06% ranging from 94,45% to 96,97%). The green dashed line represents the mean of coverage 1x across all samples (SD = 0,43) while the green solid line indicates the mean of coverage 20x across all samples (SD = 0,51).

**S2 Fig**. ECG of a negative case after NGS

**S3 Fig.** ECG of an index case
