# Supplementary material for: Genetic Analysis of Arrhythmogenic Diseases in the Era of NGS: The Complexity of Clinical Decision-Making in Brugada Syndrome
Source: PLoS One. 2015 Jul 31;10(7):e0133037. doi: 10.1371/journal.pone.0133037 (PMC4521779; doi:10.1371/journal.pone.0133037)
Supplement: S1 Table — (DOC) [file pone.0133037.s005.doc]

Supplemental table S1

| **Batch** | **Sample** | **Total Reads** | **Reads On Target** | **Enrichment fold** | **Coverage ≥ 1, (% bp)** | **Coverage ≥ 5, (% bp)** | **Coverage ≥ 10, (% bp)** | **Coverage ≥ 20, (% bp)** | **Average Depth of Coverage** | **Filtered Reads** | **Properly Paired Reads** | **Average Mapping Quality** |
| --- | --- | --- | --- | --- | --- | --- | --- | --- | --- | --- | --- | --- |
| **Batch #1** | |  |  |  |  |  |  |  |  |  |  |  |
|  | **S.1** | 6792533 | 3568805 | 11367.20 | 97.22 | 96.54 | 96.24 | 95.82 | 738.29 | 939836 | 891353 | 39.71 |
|  | **S.2** | 6852419 | 3088579 | 10791.50 | 97.26 | 96.62 | 96.29 | 95.98 | 639.44 | 893789 | 830934 | 39.43 |
|  | **S.3** | 7560766 | 3901574 | 11240.80 | 97.27 | 96.61 | 96.33 | 96.04 | 807.77 | 1173708 | 1117902 | 39.45 |
|  | **S.4** | 6532935 | 3431872 | 11640.60 | 97.40 | 96.75 | 96.42 | 96.07 | 710.26 | 919733 | 852207 | 39.63 |
|  | **S.5** | 6701805 | 2180627 | 7855.80 | 96.40 | 95.71 | 95.36 | 94.84 | 451.87 | 812790 | 764301 | 39.65 |
|  | **S.6** | 8440891 | 4595174 | 11777.00 | 97.43 | 96.90 | 96.60 | 96.25 | 950.70 | 1181375 | 1112030 | 39.95 |
|  | **S.7** | 6268377 | 3302545 | 11350.30 | 97.25 | 96.57 | 96.20 | 95.85 | 684.35 | 872810 | 813687 | 39.36 |
|  | **S.8** | 8434406 | 4307865 | 11717.20 | 97.81 | 97.24 | 96.94 | 96.50 | 893.09 | 987009 | 900384 | 39.55 |
|  | **S.9** | 7265057 | 3504004 | 10641.70 | 97.52 | 97.00 | 96.66 | 96.19 | 724.78 | 902635 | 853245 | 40.12 |
|  | **S.10** | 7094639 | 3323393 | 10940.20 | 97.70 | 96.97 | 96.60 | 96.19 | 689.16 | 1086077 | 1026578 | 40.09 |
|  | **S.11** | 5644814 | 2958852 | 11464.70 | 97.58 | 96.94 | 96.56 | 96.13 | 612.46 | 809435 | 754838 | 38.88 |
|  | **S.12** | 7826133 | 3969340 | 11828.60 | 97.20 | 96.61 | 96.35 | 95.96 | 822.10 | 988636 | 910173 | 40.13 |
|  | **Mean** | **7117898** | **3511053** | **11051.30** | **97.34** | **96.71** | **96.38** | **95.99** | **727.02** | **963986** | **902303** | **39.66** |
| **Batch #2** | |  |  |  |  |  |  |  |  |  |  |  |
|  | **S.13** | 6973714 | 3125663 | 10513.20 | 99.24 | 97.51 | 97.15 | 96.68 | 643.68 | 753169 | 691066 | 37.51 |
|  | **S.14** | 6279039 | 3177911 | 11104.90 | 98.08 | 97.28 | 96.84 | 96.39 | 655.02 | 810299 | 757859 | 36.89 |
|  | **S.15** | 5653500 | 3073150 | 11862.70 | 98.10 | 97.40 | 97.09 | 96.55 | 632.70 | 760524 | 702370 | 38.05 |
|  | **S.16** | 5124603 | 2789153 | 11822.30 | 98.05 | 97.45 | 97.04 | 96.66 | 572.42 | 905146 | 850115 | 37.62 |
|  | **S.17** | 6923472 | 3997428 | 11966.80 | 98.13 | 97.37 | 96.97 | 96.44 | 823.91 | 930958 | 869833 | 37.27 |
|  | **S.18** | 7310012 | 4107518 | 12383.70 | 98.41 | 97.70 | 97.32 | 96.97 | 846.16 | 718459 | 624738 | 36.11 |
|  | **S.19** | 5795110 | 3333047 | 12610.10 | 98.11 | 97.46 | 97.13 | 96.68 | 686.61 | 798727 | 740810 | 37.75 |
|  | **S.20** | 5467619 | 2033546 | 8971.16 | 98.15 | 97.39 | 97.03 | 96.46 | 417.75 | 483724 | 411774 | 35.28 |
|  | **S.21** | 8947524 | 3996871 | 10430.40 | 97.99 | 97.41 | 97.14 | 96.75 | 822.41 | 880162 | 802784 | 37.18 |
|  | **S.22** | 6403421 | 3541190 | 11676.10 | 98.05 | 97.42 | 96.99 | 96.53 | 728.77 | 1142918 | 1087487 | 37.83 |
|  | **Mean** | **6487801** | **3317548** | **11334.14** | **98.23** | **97.44** | **97.07** | **96.61** | **682.94** | **818409** | **753884** | **37.15** |
| **Batch #3** | |  |  |  |  |  |  |  |  |  |  |  |
|  | **S. 23** | 7643667 | 3828926 | 12028.00 | 97.76 | 97.05 | 96.64 | 95.99 | 778.49 | 652798 | 593945 | 33.11 |
|  | **S. 24** | 7811414 | 4107768 | 12970.40 | 97.90 | 97.22 | 96.86 | 96.37 | 837.79 | 647477 | 579816 | 32.72 |
|  | **S. 25** | 6129529 | 2572004 | 10408.50 | 97.82 | 97.12 | 96.66 | 96.07 | 522.25 | 657769 | 601715 | 34.55 |
|  | **S. 26** | 5650275 | 2461677 | 10264.50 | 97.59 | 96.71 | 96.20 | 95.61 | 500.24 | 640851 | 594170 | 33.99 |
|  | **S. 27** | 6787438 | 2499592 | 9503.82 | 97.76 | 96.98 | 96.55 | 95.85 | 507.06 | 570097 | 520730 | 33.88 |
|  | **S. 28** | 8303455 | 3832353 | 11577.30 | 98.02 | 97.23 | 96.81 | 96.28 | 780.07 | 592327 | 531548 | 32.88 |
|  | **S. 29** | 5909843 | 2697325 | 10791.10 | 97.65 | 96.72 | 96.25 | 95.61 | 549.07 | 712812 | 667245 | 34.74 |
|  | **S. 30** | 5458604 | 1471622 | 7034.19 | 97.42 | 96.22 | 95.59 | 94.45 | 299.73 | 463375 | 429664 | 34.61 |
|  | **S. 31** | 6358300 | 1638824 | 7059.79 | 97.94 | 96.78 | 96.22 | 95.52 | 332.94 | 514748 | 473710 | 33.80 |
|  | **S. 32** | 6253209 | 3168407 | 11909.60 | 97.95 | 97.11 | 96.71 | 96.10 | 643.04 | 711767 | 663228 | 33.97 |
|  | **S. 33** | 6746575 | 1219968 | 5201.67 | 97.72 | 96.75 | 95.95 | 94.83 | 248.14 | 404668 | 363841 | 34.11 |
|  | **Mean** | **6641119** | **2681679** | **9886.26** | **97.78** | **96.90** | **96.40** | **95.70** | **545.35** | **597154** | **547237** | **33.85** |
| **Batch #4** | |  |  |  |  |  |  |  |  |  |  |  |
|  | **S. 34** | 4699120 | 2207796 | 11916.30 | 97.88 | 96.94 | 96.44 | 95.71 | 446.26 | 621015 | 572008 | 32.29 |
|  | **S. 35** | 6237846 | 2735590 | 11690.00 | 98.15 | 97.44 | 97.00 | 96.41 | 552.90 | 611110 | 544655 | 31.95 |
|  | **S. 36** | 8232877 | 3219206 | 10697.70 | 97.39 | 96.68 | 96.23 | 95.76 | 652.57 | 715571 | 662336 | 32.60 |
|  | **S. 37** | 5645248 | 2289940 | 10835.50 | 97.91 | 97.05 | 96.57 | 95.78 | 464.11 | 591205 | 534435 | 32.12 |
|  | **S. 38** | 5757335 | 2609961 | 12068.40 | 98.00 | 97.35 | 96.91 | 96.38 | 527.40 | 552556 | 494996 | 31.92 |
|  | **S. 39** | 7067401 | 3029189 | 11314.50 | 98.14 | 97.41 | 97.05 | 96.50 | 613.00 | 777938 | 719986 | 32.76 |
|  | **S. 40** | 4935771 | 1898885 | 10323.30 | 97.92 | 97.14 | 96.72 | 95.96 | 384.02 | 474101 | 426399 | 32.11 |
|  | **S. 41** | 5662928 | 2443702 | 11467.10 | 98.01 | 97.01 | 96.55 | 95.91 | 495.25 | 534873 | 482925 | 32.27 |
|  | **S. 42** | 6765008 | 3041546 | 11607.60 | 98.12 | 97.34 | 96.98 | 96.45 | 615.81 | 663537 | 598410 | 32.08 |
|  | **S. 43** | 5813975 | 2597087 | 11956.60 | 97.85 | 97.06 | 96.56 | 95.67 | 527.35 | 592400 | 536261 | 32.44 |
|  | **S. 44** | 5340187 | 2448086 | 12114.70 | 98.08 | 97.30 | 96.73 | 95.91 | 496.55 | 583450 | 530939 | 31.61 |
|  | **S. 45** | 5529408 | 2513884 | 12320.90 | 98.14 | 97.34 | 96.86 | 96.23 | 509.35 | 385550 | 310819 | 30.48 |
|  | **Mean** | **5973925** | **2586239** | **11526.05** | **97.97** | **97.17** | **96.72** | **96.06** | **523.71** | **591942** | **534514** | **32.05** |
