# Supplementary material for: Genetic Analysis of Arrhythmogenic Diseases in the Era of NGS: The Complexity of Clinical Decision-Making in Brugada Syndrome
Source: PLoS One. 2015 Jul 31;10(7):e0133037. doi: 10.1371/journal.pone.0133037 (PMC4521779; doi:10.1371/journal.pone.0133037)
Supplement: S2 Table — (DOC) [file pone.0133037.s006.doc]

**Supplemental table S2**

| **Batch** | **Total Reads** | **Reads On Target** | **Enrichment fold** | **Coverage ≥ 1, (% bp)** | **Coverage ≥ 5, (% bp)** | **Coverage ≥ 10, (% bp)** | **Coverage ≥ 20, (% bp)** | **Average Depth of Coverage** | **Filtered Reads** | **Properly Paired Reads** | **Average Mapping Quality** |
| --- | --- | --- | --- | --- | --- | --- | --- | --- | --- | --- | --- |
| **Batch #1** | **7E+06** | 3511053 | 11051.30 | 97.34 | 96.71 | 96.38 | 95.99 | 727.02 | 963986 | 902303 | 39.66 |
| **Batch #2** | **6E+06** | 3317548 | 11334.14 | 98.23 | 97.44 | 97.07 | 96.61 | 682.94 | 818409 | 753884 | 37.15 |
| **Batch #3** | **7E+06** | 2681679 | 9886.26 | 97.78 | 96.90 | 96.40 | 95.70 | 545.35 | 597154 | 547237 | 33.85 |
| **Batch #4** | **6E+06** | 2586239 | 11526.05 | 97.97 | 97.17 | 96.72 | 96.06 | 523.71 | 591942 | 534514 | 32.05 |
| **Mean** | **######** | **3024130** | **10949.44** | **97.83** | **97.05** | **96.64** | **96.09** | **619.76** | **742873** | **684484** | **35.68** |
