# Supplementary material for: Genetic Analysis of Arrhythmogenic Diseases in the Era of NGS: The Complexity of Clinical Decision-Making in Brugada Syndrome
Source: PLoS One. 2015 Jul 31;10(7):e0133037. doi: 10.1371/journal.pone.0133037 (PMC4521779; doi:10.1371/journal.pone.0133037)
Supplement: S3 Table — Captured regions with less than 95% of their sequence covered at 20x (considered if detected in at least 5 samples). Chromosomic Region: coordinates of the region (hg19/GRCh37); Gene: HGNC gene symbol; Ensembl isoform, corresponding Ensembl gene isoform; Exon num, corresponding exon number; Num.samples, amount of samples in which region is tagged. (DOC) [file pone.0133037.s007.doc]

Supplemental table S3:

| **Chromosomic Region** | **Gene** | **Ensembl isoform** | **Exon nº** | **#samples** |
| --- | --- | --- | --- | --- |
| chr1:116268129_116268174 | *CASQ2* | ENST00000261448 | exon 7 | 48 |
| chr7:91694545_91694777 | *AKAP9* | ENST00000356239 | exon 25 | 5 |
| chr7:91706169_91706321 | *AKAP9* | ENST00000356239 | exon 29 | 5 |
| chr7:150671799_150672029 | *KCNH2* | ENST00000262186 | exon 2 | 48 |
| chr7:150655147_150655590 | *KCNH2* | ENST00000262186 | exon 4 | 48 |
| chr7:150648536_150648923 | *KCNH2* | ENST00000262186 | exon 7 | 21 |
| chr7:150646516_150647508 | *KCNH2* | ENST00000262186 | exon 9 | 48 |
| chr7:150645532_150645631 | *KCNH2* | ENST00000262186 | exon 11 | 15 |
| chr7:150644694_150644966 | *KCNH2* | ENST00000262186 | exon 12 | 48 |
| chr7:150644416_150644602 | *KCNH2* | ENST00000262186 | exon 13 | 48 |
| chr7:150643965_150644142 | *KCNH2* | ENST00000262186 | exon 14 | 22 |
| chr11:2466221_2466714 | *KCNQ1* | ENST00000155840 | exon 1 | 48 |
| chr11:2797190_2797284 | *KCNQ1* | ENST00000155840 | exon 13 | 38 |
| chr11:74165886_74168648 | *KCNE3* | ENST00000310128 | exon 1 | 48 |
| chr11:118004092_118007835 | *SCN4B* | ENST00000324727 | exon 5 | 18 |
| chr12:2224390_2224711 | *CACNA1C* | ENST00000402845 | exon 2 | 22 |
| chr12:2773991_2774156 | *CACNA1C* | ENST00000402845 | exon 35 | 5 |
