# Supplementary material for: Genetic Analysis of Arrhythmogenic Diseases in the Era of NGS: The Complexity of Clinical Decision-Making in Brugada Syndrome
Source: PLoS One. 2015 Jul 31;10(7):e0133037. doi: 10.1371/journal.pone.0133037 (PMC4521779; doi:10.1371/journal.pone.0133037)
Supplement: S4 Table — *Yes: Aborted Sudden Cardiac Death or syncope of suspected cardiac origin. A: American, EU:European; ICD: Implantable Cardioverter Defibrillator. FM: Family Members; GCR: Genetic Carrier Relatives; NGC No genetic carriers relatives. (DOC) [file pone.0133037.s008.doc]

**Supplemental table S4**

| **BrS Index Case** | **Gender** | **Age** | **Population Origin** | **Symptomatic*** | **Basal ECG** | **Drug induction study** | **EPS** | **Familiar history of SD** | **Treatment** | **FM** | **GCR** | **NGC Relatives** |
| --- | --- | --- | --- | --- | --- | --- | --- | --- | --- | --- | --- | --- |
| 1 | F | 47 | EU, Belgium | Yes | Negative | Positive Ajmaline | Positive | Positive | ICD | 2 | 1 | 1 |
| 2 | M | 38 | A, Caucasian | Yes | Positive | Not done | Unknown | Positive | ICD | - | - | - |
| 3 | M | 66 | A, Caucasian | Yes | Positive | Not done | Positive | Positive | ICD | 2 | 1 | 1 |
| 4 | M | 70 | A, Caucasian | Yes | Positive | Unknown | Positive | Negative | ICD | 1 | 0 | 1 |
| 5 | M | 30 | EU, Spain | Yes | Positive | Positive Flecainide | Positive | Negative | ICD | 1 | 1 | 0 |
| 6 | F | 48 | EU, Spain | No | Negative | Positive Flecainide | Negative | Unknown | Conservative | 4 | 1 | 3 |
| 7 | M | 44 | EU, Spain | No | Positive | Positive Flecainide | Positive | Negative | ICD | 8 | 4 | 4 |
| 8 | F | 43 | A, Caucasian | No | Negative | Positive Procainamide | Positive | Positive | Refused ICD | 5 | 4 | 1 |
| 9 | M | 74 | EU, Belgium | Yes | Negative | Positive Ajmaline | Positive | Negative | ICD | 2 | 2 | 0 |
| 10 | F | 48 | EU, Belgium | No | Negative | Positive Ajmaline | Not done | Positive | Conservative | 7 | 6 | 1 |
| 11 | F | 68 | A, Caucasian | No | Positive | Not done | Not done | Negative | Conservative | 11 | 4 | 7 |
| 12 | F | 38 | EU, Belgium | Yes | Negative | Positive Ajmaline | Negative | Negative | ICD | 9 | 3 | 6 |
| 13 | F | 52 | A, Caucasian | No | Positive | Not done | Not done | Negative | Conservative | - | - | - |
| 14 | M | 33 | A, Hispanic | Yes | Positive | Not done | Positive | Positive | ICD | - | - | - |
| 15 | M | 40 | A, Caucasian | No | Positive | Unknown | Positive | Positive | ICD | - | - | - |
| 16 | M | 41 | A, Caucasian | No | Positive | Positive Procainamide | Positive | Positive | ICD | - | - | - |
| 17 | M | 46 | A, Caucasian | Yes | Positive | Not done | Not done | Positive | Conservative | - | - | - |
| 18 | F | 64 | A, Caucasian | No | Negative | Positive Ajmaline | Negative | Unknown | Conservative | 3 | 0 | 3 |
| 19 | M | 31 | EU, Italy | No | Negative | Positive Ajmaline | Negative | Positive | Conservative | 13 | 4 | 9 |
| 20 | M | 50 | EU, Belgium | No | Positive | Positive Ajmaline | Negative | Negative | ICD | 6 | 2 | 3 |
| 21 | M | 23 | EU, Spain | Yes | Positive | Not done | Negative | Negative | ICD | 6 | 2 | 4 |
| 22 | M | 38 | EU, Spain | No | Positive | Positive Flecainide | Negative | Positive | Conservative | 3 | 1 | 2 |
